# Dioctadecyldimethylammonium bromide, a surfactant model for the cell membrane: Importance of microscopic dynamics

**DOI:** 10.1063/4.0000030

**Published:** 2020-09-22

**Authors:** V. K. Sharma, H. Srinivasan, V. García Sakai, S. Mitra

**Affiliations:** 1 Solid State Physics Division, Bhabha Atomic Research Centre, Mumbai 400085, India; 2 Homi Bhabha National Institute, Anushaktinagar, Mumbai 400094, India; 3 ISIS Facility, Science and Technology Facilities Council, Rutherford Appleton Laboratory, Didcot OX11 0QX, United Kingdom

## Abstract

Cationic lipid membranes have recently attracted huge attention both from a fundamental point of view and due to their practical applications in drug delivery and gene therapy. The dynamical behavior of the lipids in the membrane is a key parameter controlling various physiological processes and drug release kinetics. Here, we review the dynamical and thermotropic phase behavior of an archetypal cationic lipid membrane, dioctadecyldimethylammonium bromide (DODAB), as studied using neutron scattering and molecular dynamics simulation techniques. DODAB membranes exhibit interesting phase behavior, specifically showing coagel, gel, and fluid phases in addition to a large hysteresis when comparing heating and cooling cycles. The dynamics of the lipid membrane is strongly dependent on the physical state of the bilayer. Lateral diffusion of the lipids is faster, by an order of magnitude, in the fluid phase than in the ordered phase. It is not only the characteristic times but also the nature of the segmental motions that differ between the ordered and fluid phases. The effect of different membrane active molecules including drugs, stimulants, gemini surfactants, and unsaturated lipids, on the dynamical and thermotropic phase behavior of the DODAB membrane, is also discussed here. Various interesting features such as induced synchronous ordering between polar head groups and tails, sub diffusive behavior, etc., are observed. The results shed light on the interaction between these additives and the membrane, which is found to be a complex interplay between the physical state of the membrane, charge, concentration, molecular architecture of the additives, and their location within the membrane.

## I. INTRODUCTION

Lipids are one of the four building blocks of life along with nucleic acids, proteins, and carbohydrates; hence, it is of no surprise that lipid-based systems have been meticulously investigated over the last few decades. Lipids are amphiphilic molecules having a hydrophilic head and hydrophobic chain(s), which self-assemble under favorable conditions in an aqueous medium, to form micelles, liposomes, or even more complex structures. Liposomes are the closed bilayer structures encompassing an aqueous core and are surrounded by water. Lipid molecules in the bilayer structure have rich phase behavior and can exist in different polymorphic phases such as crystalline (*L*_c_), gel (*L*_β_), and fluid (*L*_α_) phases, depending on the temperature, hydration, molecular architecture, and concentration of the lipids.^1^ The physics of this rich phase behavior and associated transitions of lipids are, thus, scientifically interesting and in fact of biological relevance. The molecular arrangements of the lipids are a defining feature of these phases. The crystalline phase (e.g., coagel and subgel) has the highest order among the lamellar phases and has the highest packing density of the lipids. On the other hand, the fluid phase is highly disordered and has a large area per lipid. The crystalline/gel to fluid phase transition is associated with the transition of hydrocarbon chains from essentially an all *trans* state to a disordered state with significant *gauche* defects. Hence, it is called main transition or chain melting, and the corresponding temperature is referred to as the main phase transition temperature (*T*_m_). The fluid phase (*L*_α_) is also known as the liquid crystalline phase and closely resembles that found in cellular membranes.

**FIG. 1.**
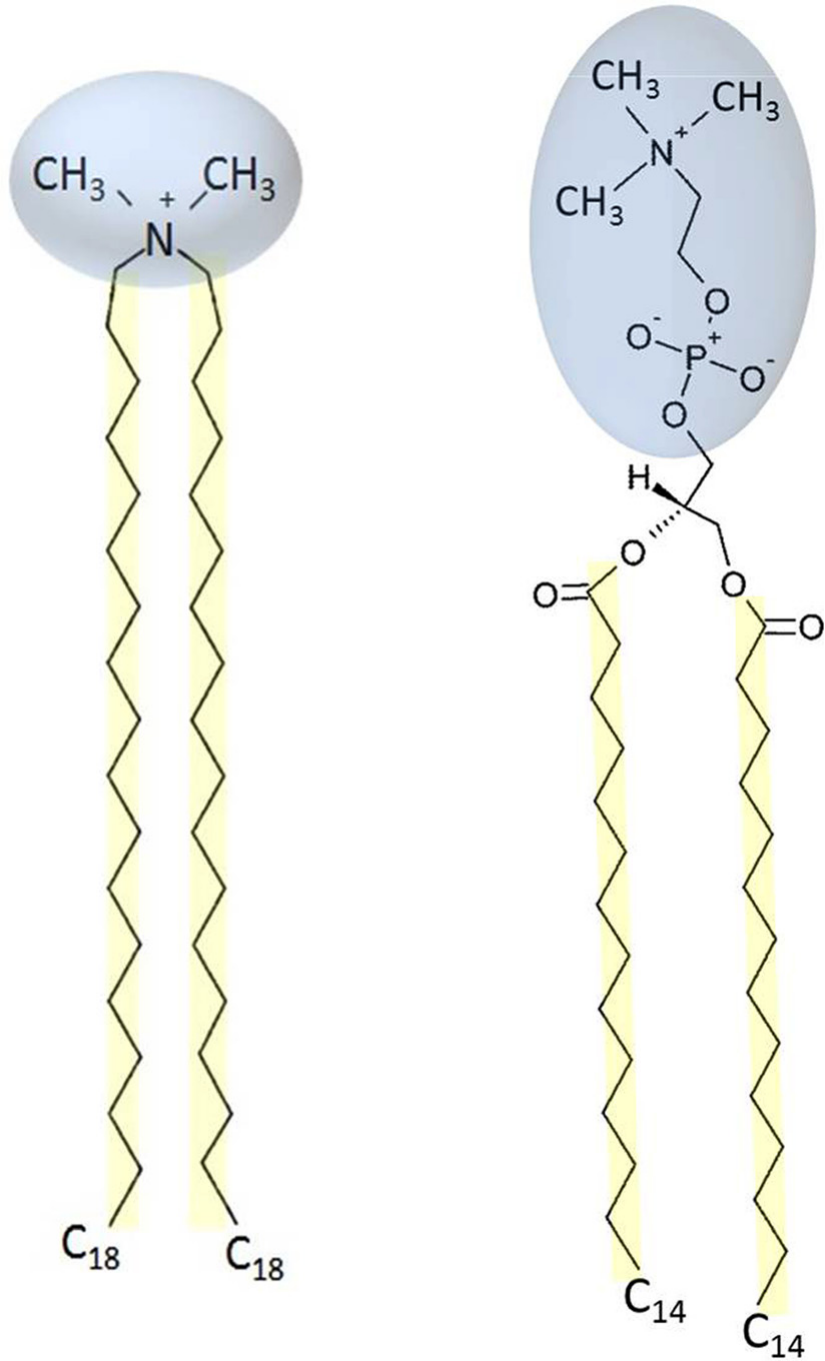
(left) Chemical structure of the cationic lipid DODAB, which has a small positively charged quaternary ammonium head group and two alkyl chains, each with 18 carbon atoms. (right) Structure of a typical zwitterionic phospholipid, 1,2-dimyristoyl-sn-glycero-3-phosphocholine (DMPC). DMPC has a relatively large head group, which has negatively charged phosphate and positively charged choline groups.

Depending on the charge, one may classify lipids as cationic, anionic, zwitterionic, and neutral lipids. Cationic lipids have attracted attention due to their potential applications in drug delivery and gene therapy.^2,3^ Nano-delivery systems based on cationic lipids are effective to transport large and negatively charged structures, such as DNA or RNA, based on electrostatic interactions.^3^ These systems have also shown stronger interaction with tumor vessels due to the over expression of negatively charged functional groups. Dioctadecyldimethylammonium bromide (DODAB) is a synthetic cationic lipid with a positively charged quaternary ammonium head group and found to form bilayer structures.^4^ DODAB [(C_18_H_37_)_2_N(CH_3_)_2_Br] has two methyl units in the headgroup and two alkyl chains of C_18_H_37_ as shown in Fig. 1. Compared to phospholipids, which are the fundamental components of the cell membrane, DODAB has a relatively smaller head group. For comparison, a typical phospholipid, namely, 1,2-dimyristoyl-sn-glycero-3-phosphocholine (DMPC), is shown in Fig. 1. DMPC has a large head group consisting of phosphate and choline groups. The positive charge on the choline and the negative charge on the phosphate make DMPC a zwitterionic lipid.

DODAB systems have been extensively studied in last three decades both from a fundamental point of view and due to their biomedical applications. The positively charged quaternary ammonium group plays a crucial role in the chemical and structural properties, in membrane stability, and interactions with oppositely charged entities of DODAB bilayer systems.^2–4^ For example, a study has revealed that the antimicrobial properties of DODAB lipids are associated with the net magnitude of charge on the DODAB lipid.^5^ The cationic surfactant and lipid, CTAB and DODAB, respectively, have shown antimicrobial characteristics by altering the cell surface charge.^6–8^ In addition, DODAB liposomes have propensity to interact with polymeric entities. DODAB has been shown to form a bilayer cover over oppositely charged polystyrene sulfate (PSS) microspheres, which can be used as an effective flocculant agent.^9^ The PSS-DODAB complex, a supramolecular biomimetic particle, has been shown to form stable PSS-DODAB-bacteriophage DNA assemblies at low DNA concentrations, that are toxic to *E*. *coli*.^10^ PSS-DODAB has also been shown to form efficient immunoadjuvants by increasing the adjuvant's colloid stability.^11,12^ Furthermore, DODAB has shown promising results in the formation of immunoadjuvants as potential candidates for the development of high efficiency vaccines.^12,13^ A study by Carvalho and Carmona-Ribeiro^14^ demonstrated the efficient drug delivery of biological macromolecules using DODAB liposomes. DODAB lipids have shown a tendency for the development of novel formulations of efficient DODAB-antimicrobial peptide (AMP) complexes.^15^ Gramicidin (Gr), an AMP, is toxic to mammalian cells, whereas, upon incorporation of Gr in DODAB bilayer fragments to form DODAB-gramicidin complexes, it has shown reduced toxicity toward eukaryotic cells and good antimicrobial features.^3^ DODAB bilayer fragments and complexes are of great interest for the study of stability and solubilization of hydrophobic drugs^12,17–21^ and the formation of liposome-gene/DNA complexes.^21–23^ DODAB bilayer fragments/liposomes have drawn attention for the understanding of various biological processes and mechanisms such as endocytosis and vesicular trafficking.^24^ Recently, DODAB:monoolein liposomes were found to be efficient non-viral vectors for gene delivery in mammalian cells.^25,26^ It has been demonstrated that cationic nanostructures based on DODAB can be used for food borne pathogens.^27^ Carmona-Ribeiro have recently summarized the versatile properties and novel applications of DODAB assemblies.^28^

Apart from the various biomedical and pharmaceutical applications, DODAB is also of fundamental interest due to its membrane mimetic properties. DODAB bilayers exhibit rich thermotropic phase behavior, in which each phase involves distinct molecular arrangements and molecular conformations over a range of concentrations.^29–32^ Over the last decade, a variety of experimental and molecular dynamics (MD) simulation techniques have been employed to understand the effect of temperature, concentration, and other parameters, on the structural, dynamical, and phase behavior of DODAX (X = Br and Cl) lipid bilayers.^29–41^ Effects of counterions on the bilayer structure, vesicle size, and the phase behavior of cationic vesicles based on dioctadecyldimethylammonium (DODA) acetate, chloride, or bromide were studied.^42^ It was found that the size of the vesicles is inversely proportional to the zeta potentials. This reflects that the size of the vesicles is controlled by the surface potential, which is determined by the nature and concentration of counterions. In the case of bromide, vesicles have the smallest zeta potential, resulting in the formation of the largest vesicles. The effect of the counterion on the phase behavior of these vesicles showed that for dioctadecyldimethylammonium acetate, there is a large hysteresis between heating and cooling; however, the extent of this hysteresis decreases as a function of counterion size and hydration. Moreover, it was shown that the smaller counterion results in a softer bilayer. Furthermore, the effect of adding a variety of membrane active molecules such as non-steroid anti-inflammatory drugs,^43^ antimicrobial peptides,^16^ gemini surfactants,^44^ unsaturated lipids,^45^ and caffeine^46^ on the properties of the DODAB bilayer has been studied and reported in the literature.

**FIG. 2.**
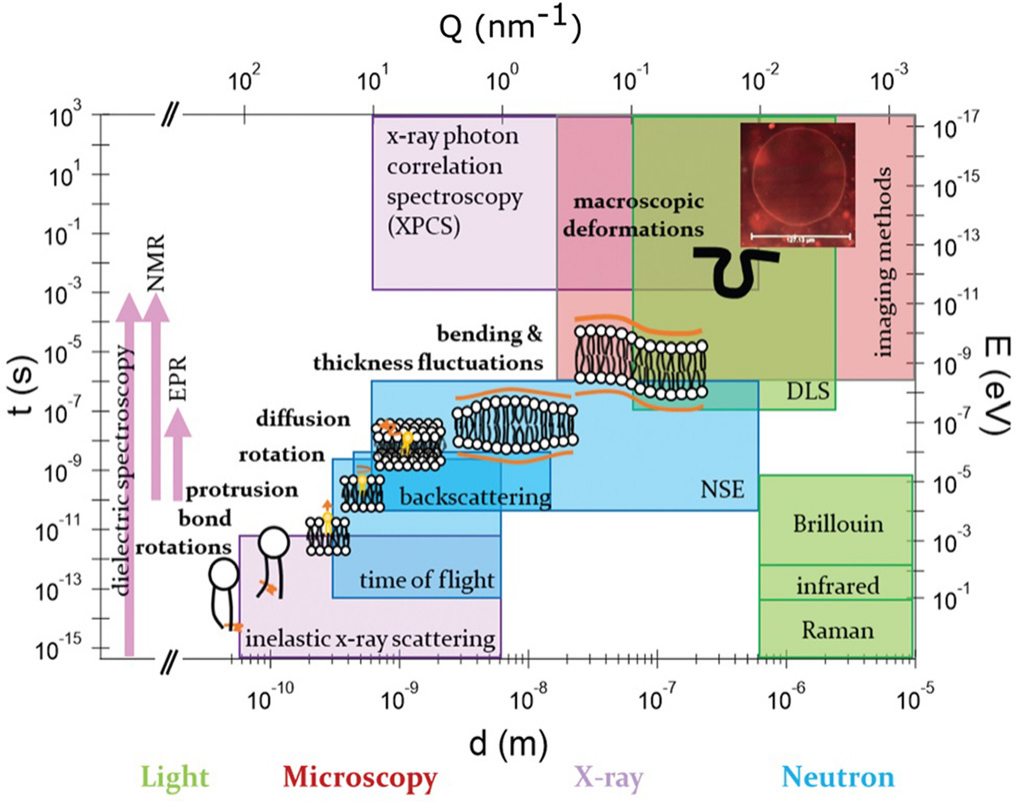
Length and time scales of the dynamical processes observed in typical lipid membranes. Included are the temporal and spatial regimes accessible by various spectroscopic methods, such as scattering techniques using different probes [e.g., neutron (blue), light (green), and x-ray (purple)], microscopy (brown), or others (NMR, EPR, and dielectric). Note that the latter can cover a wide temporal regime but are not associated with any particular spatial regime. Adapted with permission from Rickeard *et al.*, Nanoscale **12**, 1438 (2020). Copyright 2020 The Royal Society of Chemistry.

The structure and conformational rearrangements of lipid molecules in membranes are the first level at which to understand these systems. However, the resulting dynamical behavior in a membrane is key to understand many of its functions such as for processes like cell-signaling, energy transduction pathways, membrane-protein interactions, selective permeability, and cell fusion and fission, among others. Membrane dynamics also play a key role in drug encapsulation and transport if used as drug vehicles. Therefore, detailed knowledge of lipid molecular mobility in the membrane and membrane fluidity is necessary for a deep understanding of transport properties and of great relevance for pharmaceutical applications. Moreover, together with the structural and morphological properties, the molecular dynamics of lipids in different phases can provide us with a more comprehensive picture of the system's phase behavior. Lipid membranes are highly dynamic in nature and exhibit a hierarchy of dynamics, that range from individual molecular motions (such as vibrations, lateral diffusion, flip flop, and rotations) to collective modes involving many lipid molecules moving in unison (such as membrane bending motions and thickness fluctuations of the membrane).^47–53^ Altogether, the wide variety of dynamical motions span time scales over many decades, from molecular vibrations taking place in femtoseconds to flip flops over a few hours. Furthermore, the characteristic length scales of each of these motions range from Angstroms for local molecular motions to a few micrometers for macroscopic cell deformations. A schematic of the hierarchical dynamics in lipid membranes is shown in Fig. 2.

The wide variety of motions can be investigated using a range of spectroscopic methods including nuclear magnetic resonance (NMR),^53,54^ electron paramagnetic resonance (EPR),^55^ fluorescence correlation spectroscopy (FCS),^56^ dynamic light scattering (DLS),^57,58^ x-ray photo-correlation spectroscopy (XPCS),^59^ and quasi-elastic neutron scattering (QENS).^60–65^ Each method has its own advantages and limitations and can access limited spatial and temporal regimes. The accessible dynamic range of these spectroscopic methods is shown in Fig. 2. In particular, QENS is a powerful technique for studying processes occurring on length scales from a few Angstroms to hundreds of nanometers and on timescales ranging from sub-picoseconds to hundreds of nanoseconds.^66,67^ QENS techniques have been successfully used to study various lipid bilayer systems,^68–77^ providing information about both the geometry and the characteristic time scales of the molecular motions. To complement these measurements, molecular dynamics simulations can be used to provide atomistic resolution to the processes and help overcome the experimental limitations of an averaged system response, thus helping to pinpoint the role played by specific atomic groups.^78–82^ Given the computing resources available today, MD simulations can probe molecular motions in spatial and temporal domains similar to those observed in QENS and therefore give us the opportunity to directly compare and validate the physical models used to describe the QENS data.^32,83,84^ Conversely, QENS data are of importance for the validation of the force fields chosen for the MD simulations.

In this review article, we summarize the findings on the dynamical and phase behavior of a cationic DODAB lipid membrane, which serves as a model for the cell membrane. We focus on insights provided by neutron spectroscopic techniques, in particular elastic intensity scans and QENS, complemented by results from MD simulations, which provide microscopic insights on the structure and dynamics of the DODAB membrane. We will focus on the lateral and segmental motions of the lipid and furthermore consider the effects of various membrane interacting molecules such as monoolein (an unsaturated lipid), nonsteroidal anti-inflammatory drugs (NSAIDs), caffeine, and gemini surfactants on the dynamical and thermotropic phase behavior of the membranes. The results shed some light on the complex interaction between these additives and the membrane. It is found that the interaction between the additives and the lipid bilayer depends on the various factors including the physical state of the lipid bilayer, size, hydrophobicity, and pKa of the additives. Our study shows that incorporation of these additives perturbs arrangements of the lipids in the bilayer and modulates the dynamical and phase behavior of the membrane.

## II. METHODS

### A. Quasielastic neutron scattering

Neutron scattering techniques use thermal neutrons to probe the structure and dynamics of atoms or molecules in condensed matter. This is feasible as thermal neutrons have wavelengths (∼Å) that match inter-atomic spacings in materials and energies (∼meV) close to the excitation energies. In materials, motions can be broadly classified in two categories: (i) periodic motions (e.g., atomic vibrations) and (ii) stochastic motions (e.g., relaxations and diffusion). Periodic motions, oscillating with a frequency *ω*_0_, give rise to inelastic peaks at particular energy transfers, *E* = ±ℏ*ω*_0_. On the other hand, stochastic motions lead to a broadening of an elastic line measured at *E* ≈ 0, referred to as quasi-elastic broadening. Hence, the technique of quasi-elastic neutron scattering (QENS) involves the measurement of small energy transfers centered at the elastic line, typically ranging between tenths of *μ*eV to a few meV. It is a very useful technique to study atomic or molecular motions at the nanoscale. In a QENS experiment, the intensity of scattered neutrons is given by the double differential scattering cross section. It comprises two individual contributions that arise from coherent and incoherent scattering interactions between the neutrons and the sample, which can be expressed as^66^

$$\frac{d^{2}\sigma }{\mathrm{dEd}\Omega }\propto \frac{k_{f}}{k_{i}}\left[\sigma_{\mathrm{coh}}S_{\mathrm{coh}}(Q,E)+\sigma_{\mathrm{inc}}S_{\mathrm{inc}}(Q,E)\right],$$ (1)

where *S_coh_* and *S_inc_* are the coherent and incoherent scattering laws, *σ_coh_* and *σ_inc_* are the coherent and incoherent scattering cross sections, *E* is the energy transfer, and ***Q*** *=* ***k_f_*** *–* ***k_i_*** is the momentum transfer resulting from the scattering process. While the coherent scattering law contains information about pair-correlations in the system, the incoherent part is directly related to the self-correlation function. In the case of using mostly hydrogenous samples, the scattered intensity will be dominated by the incoherent scattering from the hydrogen atoms in the sample, which is due to the exceptionally high incoherent scattering cross section of hydrogen (*σ^H^_inc_* ≫ $\sigma_{\text{inc}/\text{coh}}^{\text{an}y\;\text{atom}}$). In that case, Eq. (1) can be written as

$$\frac{d^{2}\sigma }{\mathrm{dEd}\Omega }\propto \frac{k_{f}}{k_{i}}\sigma_{\mathrm{inc}}S_{\mathrm{inc}}(Q,E).$$ (2)

The incoherent scattering law, $S_{\mathrm{inc}}(Q,E)$, can provide information about the diffusion mechanism in the system as it is related to the van-Hove self-correlation function, *G_s_*(***r***,*t*), through a space-time Fourier transform,

$$S_{\mathrm{inc}}(Q,E)=\int_{-\infty }^{\infty }dt\int d^{3}re^{-i\left(\frac{E}{\hbar }t-Q.r\right)}G_{s}\left(r,t\right).$$ (3)

The van Hove self-correlation function, *G_s_*(***r***,*t*), is the probability of finding a particle at a point ***r*** in space after a time *t*, given that the particle started at the origin at *t* = 0. Therefore, based on Eqs. (2) and (3), it is clear that the measured QENS spectra can provide information about hydrogen mobility in a system. Due to the isotropic nature of the system probed, the observed scattering spectra are averaged over all the *Q*-orientations, and hence, the incoherent scattering law can be written as *S_inc_*(*Q*,*E*).

To carry out QENS experiments on liposomes and to ensure that the measured signal is primarily due to the lipids, D_2_O is used as the solvent instead of H_2_O. This reduces the scattering contribution from the solvent since deuterium has an incoherent scattering cross section, which is 40 times less than that of hydrogen. Moreover, the solvent contribution is estimated by measuring QENS spectra from D_2_O alone. Thus, the scattering signal from the lipid membrane is obtained by subtracting that of the solvent as follows:^85,86^

$$S_{\mathrm{mem}}(Q,E)=S_{\mathrm{solution}}(Q,E)-\phi S_{\mathrm{solvent}}(Q,E).$$ (4)

*S_mem_*(*Q*, *E*), *S_solution_*(*Q*, *E*), and *S_solvent_*(*Q*, *E*) are the scattering functions for the lipid membrane, total solution, and solvent, respectively. The factor, ϕ, takes into account the volume fraction of the solvent (D_2_O) in the solution.

A useful measurement in a QENS experiment is the so-called Elastic Fixed Window Scan (EFWS), where one follows the elastic intensity as a function of temperature. For a purely incoherent scattering, the elastic intensity can be used as a measure of the extent of the dynamics in the system. Furthermore, if the system undergoes a phase transition, which is accompanied by a change in its dynamical behavior, it will be reflected as a change in the elastic intensity at a given temperature in the EFWS. Therefore, the EFWS is a powerful technique to observe phase transitions associated with the dynamical changes in the system.

### B. Molecular dynamics simulations

Neutron scattering spectra result from the interactions between neutrons and an ensemble average of atoms. Thus, to obtain specific information about the dynamical behavior of specific atoms or individual components of motion, one needs to provide a description using phenomenological models. In contrast, classical molecular dynamics (MD) simulations, which provide atomistic trajectories, can be used to extract detailed dynamical information about the specific group of atoms and individual degrees of freedom in the system independently and distinctly. This information can guide the validation and improvement of phenomenological models used to analyze the QENS spectra.

In general, atomic trajectories from MD simulations are written as the sum of two contributions, center of mass (COM) motions and internal motions. Specifically, for lipids, the COM trajectory can be independently calculated and used to study the nature of lateral dynamics in the lipid bilayer. Furthermore, different structural and dynamical parameters can be calculated from MD simulation trajectories. First, the order parameter, which characterizes the structural disorder in lipid bilayer, can be calculated from the following formula:

$$S_{CH}^{i}=\frac{1}{3}\left[3\left\langle {\text{cos}}^{2}\theta_{i}\right\rangle -1\right],$$ (5)

where *θ_i_* is the angle between the *ith* CH bond vector and bilayer normal, where *i* varies between 1 to 17 starting from the headgroup to the tail of the alkyl chain. The angular brackets denote an average over all time steps and molecules in the system. Second, two important parameters that contain significant information about the dynamics of the system are the mean squared displacement (MSD) and the velocity autocorrelation function (VACF), which can be directly computed from the MD simulation trajectories, given by the following formulas:^78^

$$\begin{array}{c} \left\langle \delta r^{2}(t)\right\rangle =\frac{1}{N}\left\langle \sum_{n=1}^{N}{\left[r_{n}(t+t_{0})-r_{n}(t_{0})\right]}^{2}\right\rangle ,\\ C_{v}(t)=\frac{1}{N}\sum_{n=1}^{V}\left\langle v_{n}(t+t_{0}).v_{n}(t_{0})\right\rangle , \end{array}$$ (6)

where **r**_*n*_(*t*) and **v**_*n*_(*t*) are the position and velocity of an atom (or the molecular COM) and *N* is the total number of atoms (molecules) in the simulation box. The angular brackets denote an average over different timeorigins, *t_0_*. While the MSD or VACF can serve as key indicators of the nature of motion, the intermediate incoherent scattering function (IISF) contains more information as it essentially captures all the higher moments of displacement. The IISF can also be calculated from MD simulation trajectories as^78^

$$I(Q,t)=\frac{1}{N}\left\langle \sum_{n=1}^{N}\bar{e^{i(Q\cdot \left[r_{n}(t+t_{0})r_{n}(t_{0})\right]}}\right\rangle .$$ (7)

Here, ***Q*** is the momentum transfer, which can take values dictated by the simulation box length, *L*, such that *Q* =* 2nπ*/*L*, where *n* is an integer. The bar denotes an average over all orientations in **Q**-space to consider the isotropic nature of the system. Equations (6) and (7) can be used independently for calculating different degrees of freedom such as lateral motion and segmental motion, which can be useful to validate the phenomenological models used in the analysis of QENS data. Moreover, the IISF is directly related to the time-Fourier transform of the incoherent part of the neutron scattering law, related by the following expression:

$$S_{\mathrm{inc}}(Q,E)=\frac{1}{2\pi }\int_{-\infty }^{\infty }I(Q,t)e^{-\mathrm{iEt}/\hbar }dt.$$ (8)

Therefore, it offers a scheme to directly compare the dynamical parameters calculated from QENS and MD simulation.

## III. MICROSCOPIC DESCRIPTION OF THE PURE DODAB LIPID MEMBRANE

### A. Phase behavior

DODAB in aqueous media forms stable bilayer vesicles and exhibits rich phase behavior, which depends strongly on the concentration of the lipids and temperature. Differential scanning calorimetry (DSC) studies reveal that there are primarily four polymorphic phases in the DODAB aqueous dispersions: coagel, subgel, gel, and fluid.^36^ Below the chain melting temperature (*T*_m_), hydrated DODAB bilayers can exist in gel, subgel, or coagel phases depending on the concentration and temperature. In all these phases, the lipid chains are in an ordered, nearly all *trans* configuration but differ in their packing density, hydration, and dynamics. Further transitions are observed, such as between the subgel-gel (*T*_s_) and coagel-gel (*T*_c_) ordered phases. The main transition temperature of chain melting from the ordered (gel, subgel, and coagel) to fluid phase is found to be highly dependent on the method of preparation and the counterion present in the system.^32,34^ In particular, it has been observed that the *T*_m_ of sonicated samples is the highest, whereas that of liposomes prepared by extrusion is the lowest.^34^ In addition, significant hysteresis is observed in the phase diagram, with different temperatures being recorded in the heating and cooling cycles. At low concentrations (<1 mM), *T*_s_ and *T*_m_ are found to be ∼309 and ∼318 K^36^ when heating, but the reverse transition temperatures of gel-subgel (*T*_s_') and fluid-gel (*T*_m_') are 288 and 313 K, respectively. At slightly higher concentrations (>65 mM), there is only one transition in the heating cycle corresponding to a direct transformation from coagel to the fluid phase at a temperature, T_m_, of 326 K. While cooling, a large hysteresis is observed for the reverse transition to an intermediate gel phase, which occurs at 313 K, and a further subsequent transition from gel to coagel is observed at ∼288 K.^29,31^ The coagel is only formed at concentrations >65 mM, whereas at lower concentrations, the ordered phases are dominated by formation of subgel and gel phases. Moreover, the formation of an intermediate gel phase, which is observed only in the cooling cycle, sheds light on the interesting mechanism of the ordering induced in the headgroup and tail of the DODAB lipids. It has been suggested^31^ that this behavior is due to the nonsynchronous change between the polar headgroup and hydrophobic tails, in response to the decrease in temperature. This is mainly due to the difference in the interaction experienced between the tails and headgroups. Lipid tails mainly interact via the relatively weak van der Waals forces, whereas charged headgroups experience a strong electrostatic repulsive interaction. In the cooling cycle, as the temperature is reduced, lipid tails start ordering themselves relatively earlier compared to polar headgroups, leading to the formation of an intermediate gel phase, where the tails are ordered, but the headgroup is still disordered.

**FIG. 3.**
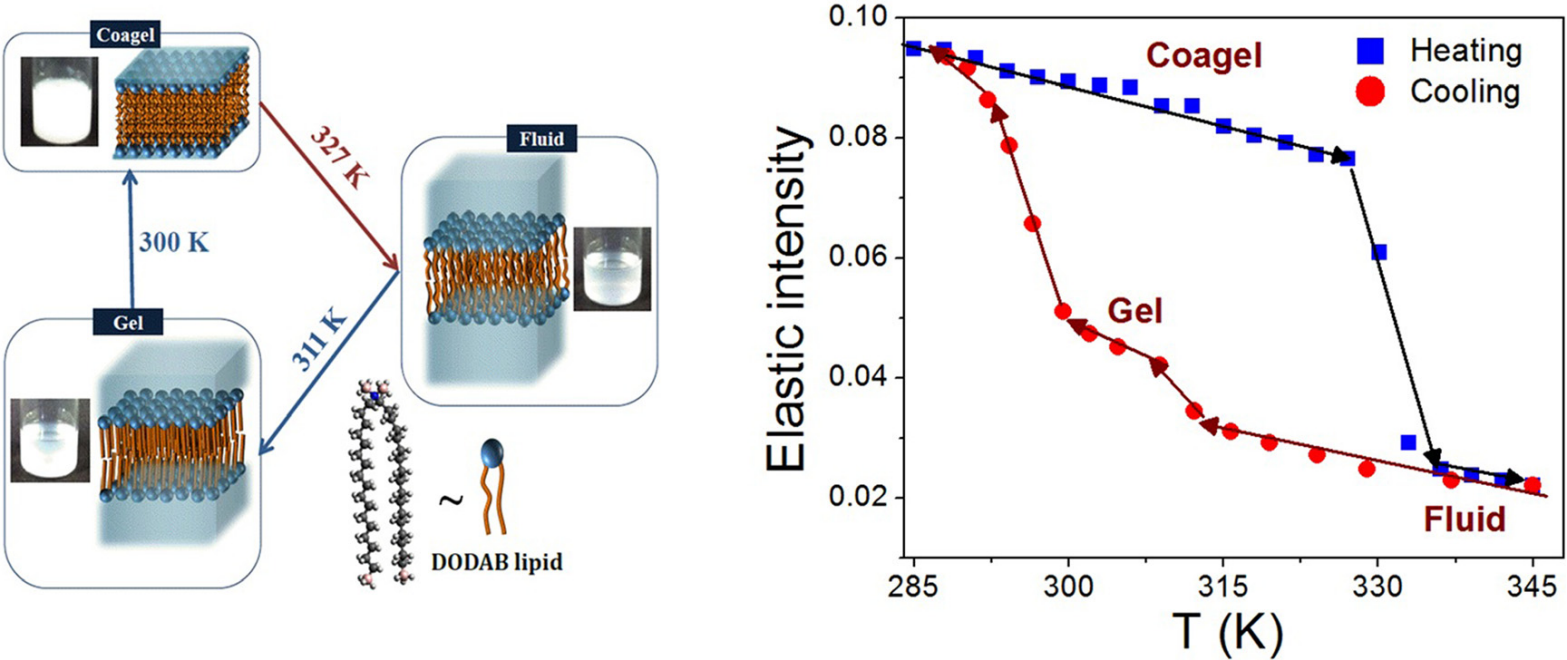
(left) Photographs of DODAB dispersion (70 mM) in different phases along with the corresponding schematics of molecular ordering in the bilayer. The water content in interlamellar spaces decreases progressively as one goes from fluid to gel and from gel to coagel phases. (right) *Q*-averaged elastic intensity scan for DODAB vesicles in the heating and cooling cycles. Adapted from Dubey *et al.*, Sci. Rep. **8**, 1862 (2018). Copyright 2018 licensed under a Creative Commons Attribution (CC BY) license.

Small angle x-ray scattering (SAXS) has been used to study the effect of the concentration on the liposome structure. In the low concentration regime (<1 mM), DODAB liposomes mostly form unilamellar vesicles (ULVs) in all the phases. However, in the concentration range of 1–65 mM, there is mixture of ULV and multilamellar structures (MLSs) in the ordered phases (gel and subgel), which transform into ULVs in the fluid phase.^36^ Above 65 mM, there is only one transition in the heating cycle corresponding to the coagel-fluid transition at T_m_, below which the Bragg peak observed with SAXS indicates that the liposomes are predominantly in MLSs.^36^ Above T_m_, the Bragg peak vanishes, suggesting the absence of any long range ordering and formation of ULVs in the fluid phase. The thickness of the bilayer in the MLS estimated from the observed Bragg peak is around ∼37 Å. The extended chain length of DODAB molecules is ∼25 Å, yielding a bilayer thickness of 50 Å. The significantly smaller thickness observed (37 Å) is mostly due to strong interdigitation of the alkyl chains from the layers in the bilayer.^36^ Such interdigitation of the chains has also been observed previously by experiments^87^ and in MD simulations of the DODAB bilayer in the ordered phase.^37^ Actual photographs of DODAB dispersion (70 mM) in different phases along with the schematic of molecular ordering of the bilayer are shown in Fig. 3.

Fourier Transform Infrared (FTIR) experiments were carried out to observe the order and packing density of the bilayer in various phases.^29^ The packing density of lipids in the bilayer is the highest in the coagel phase followed by the subgel and gel phases, with the fluid phase being the most loosely packed state.^29^ The packing density of lipids in each of these phases is directly associated with the degree of *gauche* defects in the lipid tail, which was found to be significant only in the fluid phase. It has been observed that in the gel phase, the alkyl chains organize themselves in a hexagonal 2D lattice with NMR measurements, indicating that there might be a mild rotational degree of freedom in the bilayer.^30^ However, upon further cooling, the transition to the subgel phase takes place in which alkyl chains lose the rotational degree of freedom and arrange themselves in a triclinic 2D lattice.^30^ In the coagel phase, the lipid bilayer is essentially similar to that in the subgel phase, but with strongly dehydrated headgroups as observed from FTIR measurements.^29,31^ The Cryo-TEM measurement on the gel phase reveals that the morphology of the vesicles is dominated by curled membranes; however, in the subgel phase, sharp features of facetted ULV is observed.^31^ These morphological changes can be attributed to the alkyl chain packing density in these phases. While both the gel and coagel phases showed an almost all *trans* configuration, they both differ in their hydration of the headgroup. The hydration of the headgroup in the gel phase indicates that the headgroups remain disordered although the lipid tails are ordered. This is due to the nonsynchronous change in the polar head groups and lipid tails during the cooling cycles.

The presence of hysteresis in the phase behavior of DODAB is in stark contrast to a typical zwitterionic phospholipid like DMPC in which no hysteresis in the main transition is observed.^85^ Moreover, unlike DODAB, there is no nonsynchronous change in the headgroup and tails. In both cases, this could be attributed to the difference in the molecular architecture and the polarity of the two lipids. DMPC is zwitterionic and has a relatively large head group, whereas DODAB has positively charged and relatively small headgroups. The smaller size and strong electrostatic interaction allow asynchronous transformation in the head and tail regions of the lipid, leading to a richer phase behavior compared to phospholipids.

Elastic fixed window scans in quasi-elastic neutron scattering measurements serve to characterize dynamical transitions occurring alongside structural phase transitions, and it is useful to compare with DSC data. Measurements on a 70 mM DODAB showed a sharp endothermic peak at 327 K in the heating cycle and a sharp exothermic peak at 311 K in the cooling cycle. An additional broad exothermic hump spanning 284–292 K was also observed in the cooling cycle.^33^ As discussed earlier, the endothermic peak at 327 K in the heating cycle corresponds to the coagel-fluid phase transition (T_m_), whereas in the cooling cycle, the exothermic peak at 311 K and broad hump at 284–292 K correspond to the fluid-gel and gel-coagel transitions, respectively. DSC measurements in the second heating cycle showed similar thermograms as observed in the first heating cycle. This indicates that the state at the lowest temperature of the cooling cycle is actually the coagel phase.^29^ EFWS data measured on the same sample are shown in Fig. 3. The elastic intensity decreases sharply at 327 K in the heating cycle, and two sharp changes in the slope are observed at 311 and 300 K during the cooling cycle, observations which are consistent with the DSC results. These transitions correspond to coagel−fluid during the heating cycle and fluid−gel and gel−coagel during the cooling cycle, respectively. EFWS measurements showed that these transitions are associated with the change in the microscopic dynamics of the system, suggesting that the fluid phase is the most dynamically active and the coagel phase is the least. The dynamics of lipids in each of these phases can be directly related to the lipid packing density. Evidently, the coagel phase, having the highest lipid packing density, shows the lowest dynamical activity, followed by a slightly loosely packed gel phase. In the fluid phase, which has very loose packing and significant gauche defects, the lipids are highly dynamic. As pointed out previously, it must be noted that the bilayer is in multilamellar structures (MLSs) in the coagel phase, while it transforms into a unilamellar vesicle (ULV) in the fluid phase.^36^

### B. Dynamical description of DODAB from QENS experiments

EFWS measurements showed that during the phase transitions (coagel to fluid and fluid to coagel via the gel phase), the microscopic dynamics of the DODAB membrane change. In order to get more detailed insights into the dynamical processes in each of these phases, QENS experiments were carried out at 315 and 345 K in the heating cycles, where DODAB bilayers are in the coagel and fluid phases, respectively, and at 330 and 315 K (fluid phase) and at 308 K (gel phase), in the cooling cycle. The two QENS measurements at 315 K, namely, during heating and cooling cycles, allow us to compare the system dynamics in the fluid (during cooling) and coagel (during heating) phases at the same temperature.

**FIG. 4.**
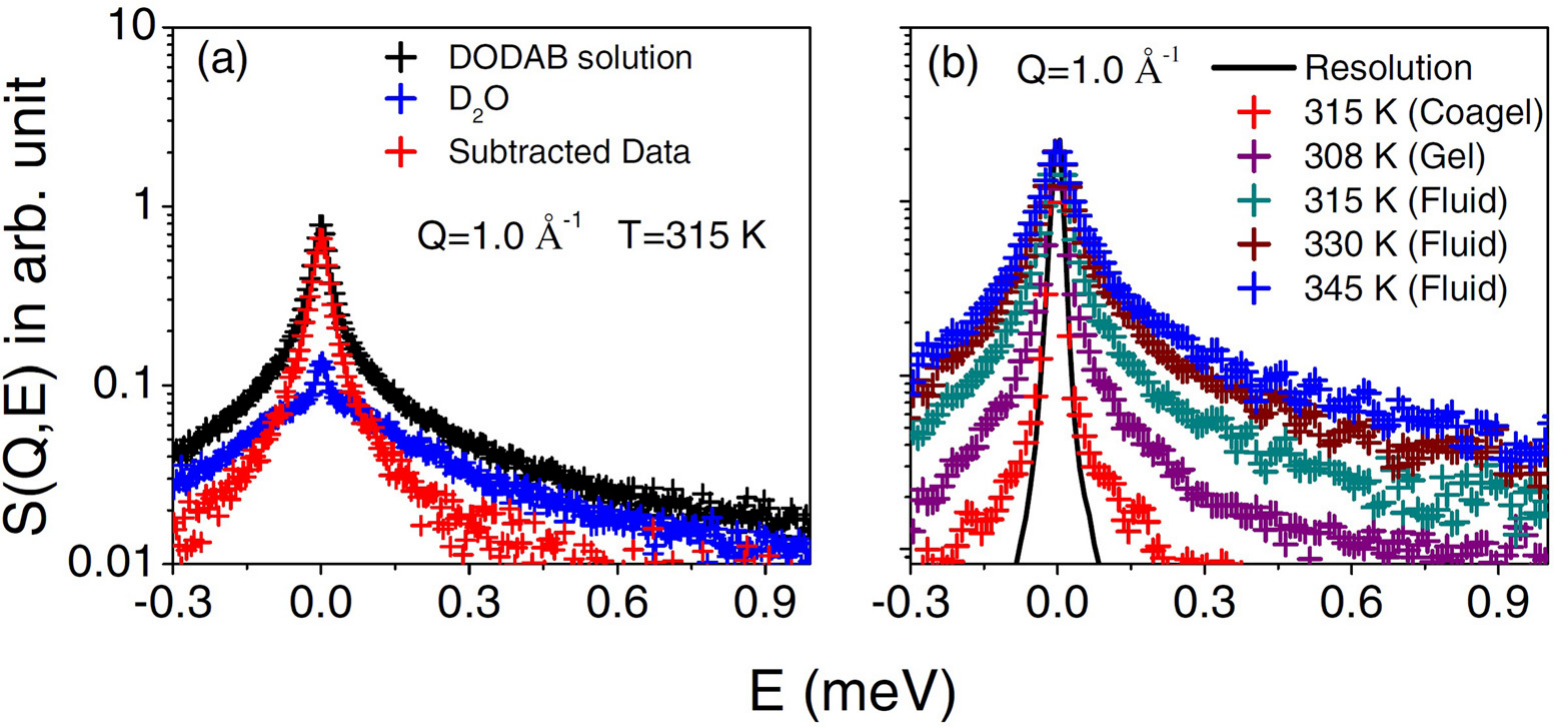
(a) QENS spectra for DODAB solution and D_2_O at 315 K at *Q* = 1.0 Å^−1^. Solvent contribution has been subtracted using Eq. (4), and contribution from the DODAB membrane is also shown. (b) D_2_O subtracted QENS spectra for the DODAB lipid membrane at different temperatures at *Q* = 1.0 Å^−1^. The instrumental resolution as measured using standard vanadium is also shown by a solid line. Spectra are normalized to the peak amplitude of the instrument resolution, which enables a direct comparison of the quasi-elastic broadening. Adapted from Dubey *et al.*, Sci. Rep. **8**, 1862 (2018). Copyright 2018 licensed under a Creative Commons Attribution (CC BY) license.

QENS spectra for DODAB solution in D_2_O (in cooling cycle) and pure solvent (D_2_O) at 315 K are shown at a representative *Q* value of 1.0 Å^−1^ in Fig. 4(a). The contribution from the solvent D_2_O has been subtracted using Eq. (4), and the resultant spectra for the DODAB bilayer in the coagel phase at 315 K (heating cycle), in the fluid phase at 345, 330, and 315 K (cooling cycle), and in the gel phase at 308 K (cooling cycle) are also shown in Fig. 4(b). The instrument resolution, as measured using a standard vanadium sample, is also shown in Fig. 4(b). To enable a direct comparison of the quasi-elastic broadening, QENS spectra at different temperatures/phases are normalized to the peak intensity of the instrument resolution. It is clear from Fig. 4 that in all the phases, significant quasi-elastic broadening is observed over the instrument resolution, suggesting the presence of stochastic dynamics associated with the DODAB lipid. It is evident that the fluid phase, at 315 K, shows a much larger broadening than the coagel phase at the same temperature, indicating that motions are significantly constrained in the latter phase.

The measured QENS spectra contain information about the dynamics of the DODAB lipid, and two distinct motions are expected to contribute, namely, the lateral motion of the whole lipid within the leaflet and localized segmental motions of the lipids.^33^ It is reasonable to consider that these two motions are not coupled to each other, in which case the effective scattering law for the membrane can be written as

$$S_{\mathrm{mem}}(Q,E)=S_{\mathrm{lat}}(Q,E)⊗S_{\mathrm{seg}}(Q,E),$$ (9)

where *S_lat_*(*Q*,*E*) and *S_seg_*(*Q*,*E*) are the scattering functions corresponding to lateral and segmental motions of DODAB molecules, respectively. The lateral motion of the lipid within the leaflet is of key interest since it plays an important role in various physiologically relevant membrane processes including cell signaling, membrane trafficking, and cell recognition. However, the model description of the lateral motion of the lipids is not fully agreed upon in the literature. Different models such as Fickian diffusion,^64^ ballistic flow like motion,^63,83^ localized translational motion of lipids in a cylindrical volume,^88^ and sub-diffusive motions^32,84^ have been used to describe the lateral motion of the lipid. The corresponding scattering laws for each of these models are very different.

We begin by considering the simplest phenomenological model, Fickian diffusion, which is the lowest order approximation to any diffusive motion in thermal equilibrium. Furthermore, recent QENS results indicate that lateral motions follow Fickian diffusion at least a distance larger than the lipid diameter.^64^ In this case, the scattering law for lateral motion can be written as

$$S_{\mathrm{lat}}(Q,E)=L_{\mathrm{lat}}(\Gamma_{\mathrm{lat}},E),$$ (10)

where *L*_lat_(Γ_lat_,*E*) is a Lorentzian with a half-width at half-maximum (HWHM), Γ_lat_, which corresponds to the relaxation rate of the lateral motion in the system.

The second motion is the segmental motion of the lipids, which is localized in nature. This gives rise to an elastic contribution in the scattering law due to the finite probability of finding the atoms within the volume observed even after sufficiently long times. The scattering law for segmental motions can be written as

$$S_{\mathrm{seg}}\left(Q,E\right)=A\left(Q\right)\delta \left(E\right)+\left(1-A\left(Q\right)\right)L_{\mathrm{seg}}\left(\Gamma_{\mathrm{seg}},E\right).$$ (11)

The first term in the above equation represents the elastic part. The second term is the quasi-elastic component, which is approximated with a single Lorentzian function, *L_seg_*(Γ_*seg*_,*E*), with a half-width at half-maximum (HWHM), Γ_*seg*_, which is inversely proportional to the characteristic time of segmental motion. The contribution of the elastic scattering out of the total scattering is called the elastic incoherent structure factor (EISF). Therefore, *A(Q)* in Eq. (11) is nothing but the EISF, which provides information about the geometry of the segmental motion.

Considering both dynamical modes, the effective scattering law for DODAB membranes [Eq. (9)] can be written as a convolution of both the components, such that

$$S_{\mathrm{mem}}(Q,E)=A(Q)L_{\mathrm{lat}}(\Gamma_{\mathrm{lat}},E)+(1-A(Q)L_{\mathrm{tot}}(\Gamma_{\mathrm{lat}+\mathrm{seg}},E).$$ (12)

In this equation, the narrower Lorentzian, *L_lat_*(Γ_*lat*_,*E*), is attributed to the slower lateral motion of DODAB with a HWHM, Γ_*lat*_, and the broader Lorentzian, *L_tot_* (Γ_*lat+seg*_,*E*) is ascribed to both lateral and segmental motions of DODAB, whose HWHM is Γ_*lat+seg*_ = Γ_*lat*_+Γ_*seg*_.

**FIG. 5.**
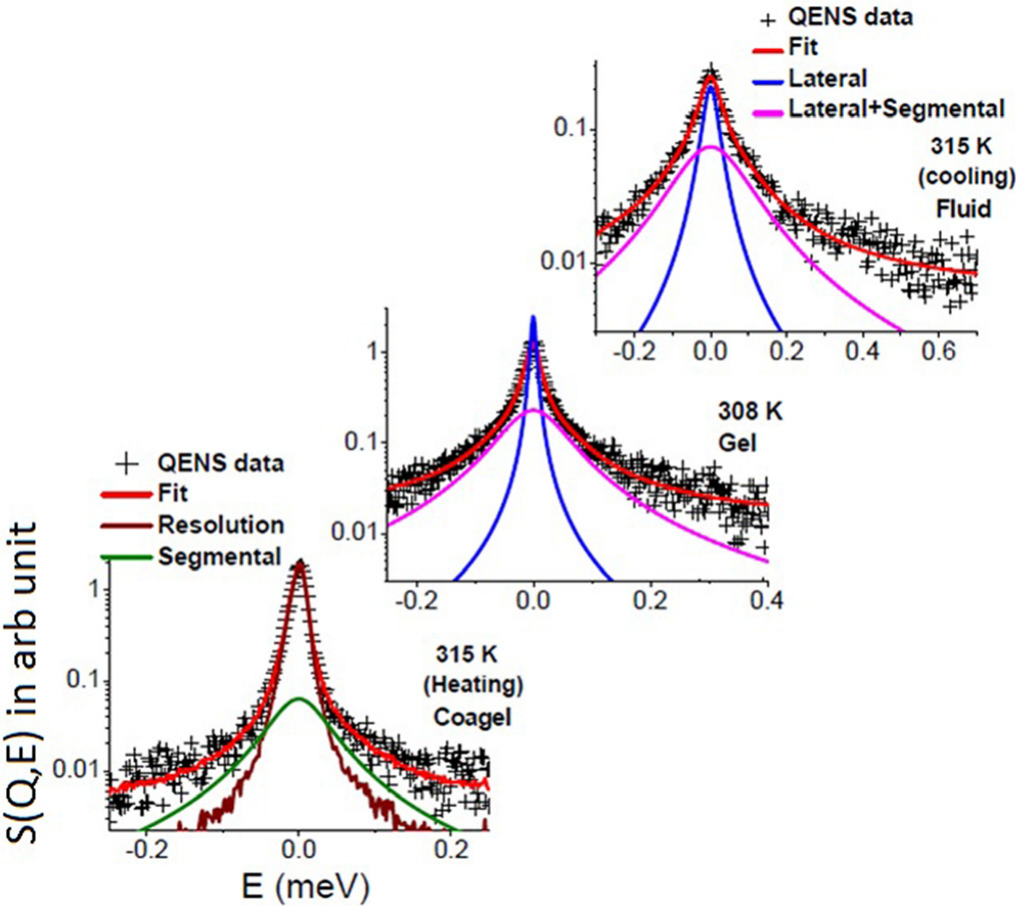
Typical fitted QENS spectra for the DODAB membrane in the coagel phase at 315 K (heating), in the gel phase at 308 K, and in the fluid phase at 315 K (cooling) at *Q* = 1.41 Å^−1^. Adapted from Dubey *et al.*, Sci. Rep. **8**, 1862 (2018). Copyright 2018 licensed under a Creative Commons Attribution (CC BY) license.

For the sample at a concentration of 70 mM, Eq. (12) could describe the measured QENS spectra quite well in both gel and fluid phases, suggesting that both lateral and segmental motions of DODAB lipids are present in these phases. However, in the case of the coagel phase, Eq. (11) that includes only segmental motions is sufficient to describe the data. This is consistent with the fact that in the coagel phase, lipid molecules are less hydrated and more densely packed (Fig. 3), resulting in lateral motions that are too slow to be observed in the timescale of the IRIS spectrometer. Example fitted spectra in coagel (315 K, heating), gel (308 K), and fluid (315 K, cooling) phases at a representative *Q* value of 1.41 Å^−1^ are shown in Fig. 5.

#### 1. Lateral motion

**FIG. 6.**
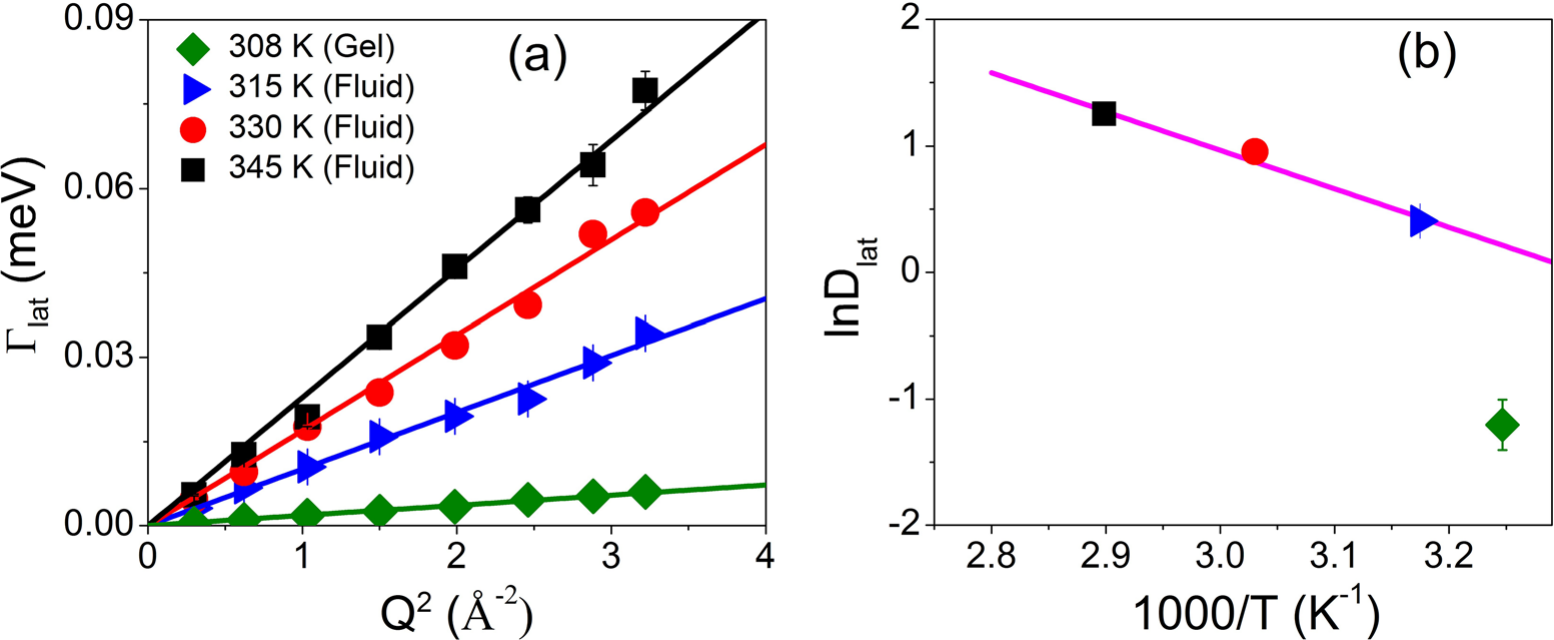
(a) Variation of HWHM of Lorentzian corresponding to lateral motion, Γ_*lat*_, with *Q*^2^ in the gel and fluid phases. The solid lines correspond to the Fickian description. (b) Variation of *D_lat_* with temperature. The solid line corresponds to the Arrhenius description. Adapted from Dubey *et al.*, Sci. Rep. **8**, 1862 (2018). Copyright 2018 licensed under a Creative Commons Attribution (CC BY) license.

As mentioned above, the lateral motion of DODAB lipids is observed in both the gel and fluid phases. The *Q* dependence of Γ_*lat*_ in the fluid phase at 315, 330, and 345 K and in the gel phase at 308 K is shown in Fig. 6(a). It is evident that in both phases, Γ_*lat*_ varies linearly with *Q^2^*, as expected for a continuous diffusion obeying Fick's law $\Gamma_{\mathrm{lat}}(Q)=D_{\mathrm{lat}}Q^{2}$. The variation of the lateral diffusion constant, *D_lat_*, with temperature is shown in Fig. 6(b). It is evident that in the fluid phase, *D_lat_* follows an Arrhenius law, $D_{\mathrm{lat}}=D_{\mathrm{lat}}^{0}\;\text{exp}\;\left(E_{a}/k_{B}T\right)$, with an activation energy (*E*_a_) of 6.12 kcal/mol. This is similar to that observed for the DMPC membrane in the fluid phase (*E_a_* = 7.4 kcal/mol).^89^ In the gel phase, *D_lat_* is found to be 0.3 (±0.1) × 10^−6^ cm^2^/s at 308 K, which is almost an order of magnitude smaller than that in the fluid phase. In the gel phase, alkyl chains are more ordered and densely packed than in the fluid phase,^90^ which restricts the lateral movement of the lipid molecules. Similar results are found for other phospholipids^61^ where in the ordered phase, the lateral diffusion coefficient is slower by at least an order of magnitude.

#### 2. Segmental motion

**FIG. 7.**
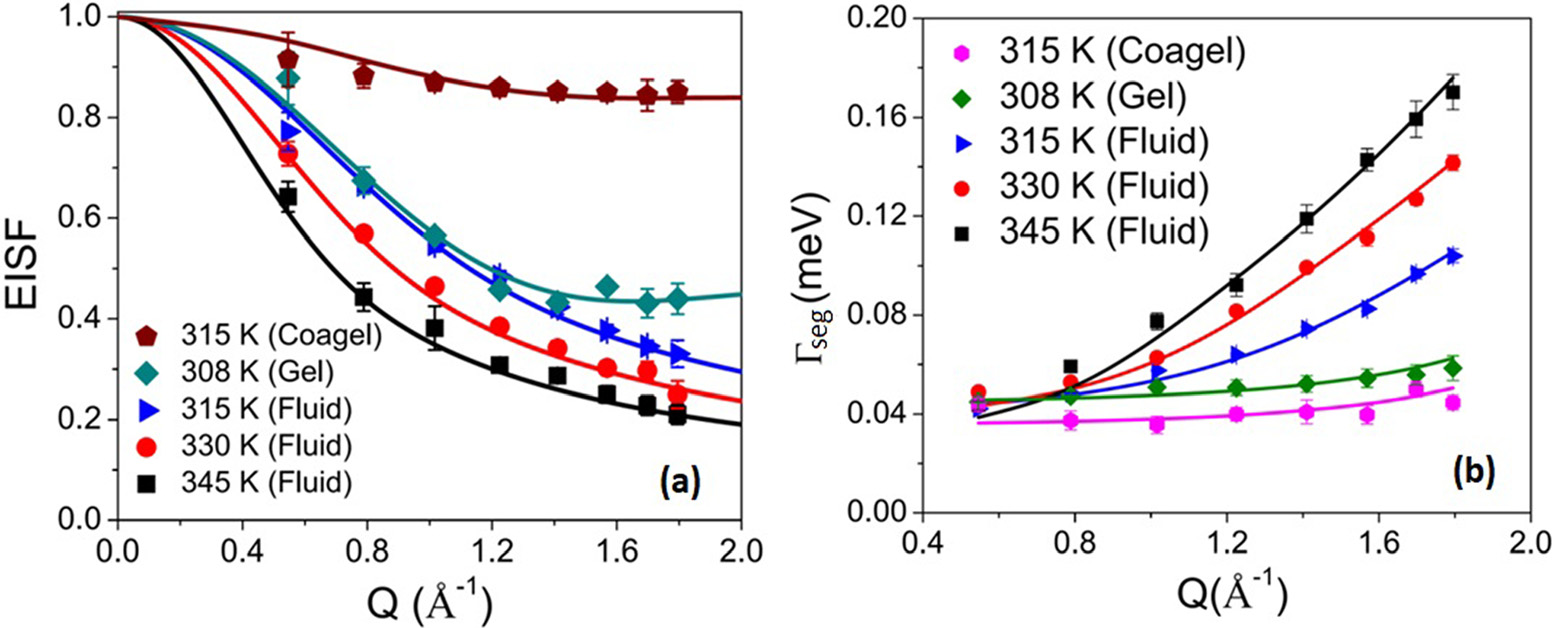
Variation of (a) the EISF and (b) HWHM for the segmental motions of DODAB lipid in the coagel, gel, and fluid phases. The solid lines represent the least squares fits assuming the models described in the text. Adapted from Dubey *et al.*, Sci. Rep. **8**, 1862 (2018). Copyright 2018 licensed under a Creative Commons Attribution (CC BY) license.

The *Q* dependences of the EISF, *A*(*Q*), and the HWHM, Γ_seg_(Q), for segmental motion at all the phases are shown in Figs. 7(a) and 7(b), respectively. It is evident that the coagel phase has the highest EISF, suggesting it to be the most ordered phase, as was observed from FTIR.^29^ Furthermore, the *Q* dependence of HWHM indicates that segmental motion in the fluid phase is very different from that in the gel and coagel phases. Noting that a DODAB molecule has two methyl units in the head group and two octadecyl alkyl chains (C_18_H_37_) in the tail, the resulting scattering law for segmental dynamics would be a combination of head group motion (threefold reorientation) and motion of the alkyl chain (lipid tail). In the ordered phase (coagel and gel), lipid molecules are tightly packed, and alkyl tails are predominantly in an all-*trans* conformation. Therefore, it would be suitable to describe the alkyl chain motion in these states with a uniaxial rotational diffusion (URD) along their axes on a circle with a radius of gyration *a*. At a given temperature, not all hydrogen atoms in the alkyl chain will be mobile, and thus, only a fraction, *p_x_,* will take part in the dynamic response. In such a scenario, the generalized scattering law for segmental motion for the gel and coagel phases can be written as^33^

$$S_{\mathrm{seg}}^{\mathrm{gel}/\mathrm{coagel}}\left(Q,E\right)=A_{\mathrm{gel}/\mathrm{coagel}}\left(Q\right)\delta \left(E\right)+\frac{1}{\pi }\left[\frac{2P_{h}}{3}\left[1-j_{0}\left(Qb\right)\right]\frac{3\tau_{MG}}{9+\tau_{MG}^{2}E^{2}}+p_{x}P_{t}\sum_{n=1}^{N_{S}-1}B_{n}\left(Qa\right)\frac{\tau_{n}}{1+\tau_{n}^{2}E^{2}}\right],$$ (13)

with

$$B_{n}(Qa)=\frac{1}{N_{s}}\sum_{i=1}^{N_{s}}j_{0}\left(2Qa\;\text{sin}\;\frac{\pi i}{N_{s}}\right)\text{cos}\;\frac{2\pi ni}{N_{s}}$$

and $\tau_{n}^{-1}=2\tau^{-1}\;{\text{sin}}^{2}(\frac{n\pi }{N_{s}})$. Here, *j_0_* is the spherical Bessel function of the zeroth order, *N_s_* (*N_s_* > 6) is the number of equivalent sites equally distributed on a circle, and *τ* is the average time spent on a site between two successive jumps. In this case, the rotational diffusion constant *D_r_* can be written as

$$D_{r}=\frac{2}{\tau }\;{\text{sin}}^{2}\left(\frac{\pi }{N_{s}}\right).$$

In Eq. (13), *A*_gel/coagel_ (*Q*) is the EISF in these phases and is given by^33^

$$A_{\mathrm{gel}/\mathrm{coagel}}(Q)=\frac{P_{h}}{3}[1+2j_{0}(Qb)]+P_{t}(1-p_{x})+P_{t}\left[\frac{p_{x}}{N_{s}}\sum_{i=1}^{N_{s}}j_{0}\left(2Qa\;\text{sin}\;\frac{\pi i}{N_{s}}\right)\right],$$ (14)

where *P_h_* is the fraction of hydrogen atoms in the head group and *P_t_* is the fraction of hydrogen atoms in the alkyl chain. For the DODAB molecule [(C_18_H_37_)_2_N^+^(CH_3_)_2_Br^−^], *P_h_* and *P_t_* are equal to 6/80 and 74/80, respectively. Here, *b* is the H-H distance (1.8 Å) in the methyl group and *τ_MG_* is the mean residence time of a hydrogen atom in a methyl group. Satisfactory fits of the EISF to Eq. (14) are shown in Fig. 7, for the coagel and gel phases. In the coagel phase, *p_x_* and *a* are found to be 0.15 and 1.7 ± 0.1 Å, respectively. This indicates that only 15% of the hydrogen atoms in the alkyl chains of DODAB molecules take part in the uniaxial rotation with a radius of gyration of 1.7 Å, which is very close to the average distance of hydrogen atoms from the chain axis. In the gel phase, *p_x_* and *a* are found to be 0.64 and 1.8 ± 0.1 Å, respectively. Therefore, the fraction of mobile hydrogens increases sharply from 15% to 64% in the gel phase compared to the coagel phase, which is in agreement with the fact that the packing density of alkyl chains decreases on going from the coagel to the gel phase.^29,31^

The rotational diffusion coefficient *D_r_* and τ_MG_ can be obtained for both phases by a least squares fitting of Eq. (13) to the experimental HWHM. *D_r_* is 5.3(±0.2) × 10^10^ s^−1^ and 6.8 (±0.3) × 10^10^ s^−1^ for the coagel (315 K, heating cycle) and gel phases (308 K, cooling cycle), respectively. Mean residence times for hydrogen in the methyl group, τ_*MG*_, are found to be 6.7 ± 0.3 and 5.2 ± 0.2 ps in the coagel and gel phases, respectively. In the coagel phase, the slower rotational diffusion for the alkyl chains and the higher residence times of hydrogens in the head groups can again be ascribed to the lower hydration level and the denser packing of the lipid molecules in comparison to the gel phase.^29–31^

In the fluid phase, DODAB molecules are disordered and loosely packed^29^ having a significant number of *gauche* defects along the alkyl chain. These structural changes result in alkyl chains being able to undergo a variety of motions, including chain reorientations, conformational changes, bending, and stretching. The superposition of these motions can be approximated by a simple model in which the H atoms of the CH_2_ units perform localized translational diffusion (LTD) confined within the spheres. This is corroborated by the observed *Q* dependence of Γ_seg_(*Q*) in the fluid phase, which shows a finite non-zero value in the zero-*Q* limit and monotonically increases with *Q*, a typical signature of the LTD model. In fact, the LTD model was used successfully to describe the dynamics of alkyl chains in various molecular aggregates, such as micelles, vesicles, and microemulsions.^91–97^ Due to the flexibility of the alkyl chains, the hydrogen atoms along the alkyl chain may not all have the same diffusivities and the same confining sphere sizes. The distribution of these parameters along the chain length can be modeled in different ways, including a linear variation, and Gaussian, lognormal.^32,98,99^ For surfactants, a linear distribution has been used to describe the dynamics in various molecular aggregates, such as micelles, vesicles, and lipid membranes.^85,91–97^ To begin with, we have assumed the same linear distribution to describe the segmental motion of the DODAB lipid in the fluid phase. In this model, the CH_2_ unit closest to the head group diffuses in a sphere of the smallest radius and the lowest diffusivity. The size of the sphere and the associated diffusivity increase linearly as we progress toward the tail of the alkyl chain. The largest radius and diffusivity are associated with the carbon atoms at the very end of the chain. Considering threefold rotation for the methyl units in the head group and the LTD model for the alkyl chain, the generalized scattering law for segmental motion of the DODAB lipid in the fluid phase can be written as^33^

$$S_{\mathrm{int}}\left(Q,E\right)=\frac{1}{80}\left[\begin{array}{c} \left\{2\left(1+2j_{0}(Qb)\right)+\frac{74}{N_{C}}\sum_{i=1}^{N_{C}}{\left[\frac{3j_{1}\left(QR_{i}\right)}{QR_{i}}\right]}^{2}\right\}\delta \left(E\right)\\ +\frac{1}{\pi }\left\{4\left(1-j_{0}(Qb)\right)\frac{3\tau_{MG}}{9+E^{2}\tau_{MG}^{2}}+\frac{74}{N_{C}}\sum_{i=1}^{N_{C}}\sum_{\left\{l,n\right\}\neq \left\{0,0\right\}}\left(2l+1\right)A_{n}^{l}\left(QR_{i}\right)\frac{{\left(x_{n}^{l}\right)}^{2}D_{i}/R_{i}^{2}}{{\left({\left(x_{n}^{l}\right)}^{2}D_{i}/R_{i}^{2}\right)}^{2}+E^{2}}\right\} \end{array}\right],$$ (15)

where *N_C_* = 18 is the total number of the CH_2_ units in the alkyl chain. The EISF for the fluid phase can be written as

$$\mathrm{EISF}=\frac{1}{80}\left[2\left[1+2j_{0}(Qb)\right]+\frac{74}{N_{C}}\sum_{i=1}^{N_{C}}{\left[\frac{3j_{1}\left(QR_{i}\right)}{QR_{i}}\right]}^{2}\right],$$ (16)

where *R*_i_ and *D*_i_ are the radius and the diffusion coefficient corresponding to the *ith* site of the lipid tail and can be written as

$$R_{i}=\frac{i-1}{N_{C}-1}\left[R_{\text{max}}-R_{\text{min}}\right]+R_{\text{min}}$$

and

$$D_{i}=\frac{i-1}{N_{C}-1}\left[D_{\text{max}}-D_{\text{min}}\right]+D_{\text{min}}.$$

This model has been used to describe the EISF and Γ_seg_ in the fluid phase, and their profiles are shown by the solid lines in Fig. 7. It is evident that this model describes the obtained EISF and HWHM for DODAB lipids in the fluid phase quite satisfactorily. The values of *R*_min_ and *D*_min_ are found to be unrealistically small for both the systems, indicating the negligible movement of the hydrogen atoms in the first carbon position held by the head-group. At 315 K, *R_max_* and *D_max_* are found to be 3.3(±0.3) Å and 12.8 (±0.5) × 10^−6^ cm^2^/s. With increasing temperature, the values of *R_max_* and *D_max_* also increase and reach up to 5.4 (±0.3) Å and 26.1 (±0.5) × 10^−6^ cm^2^/s at 345 K.

In summary, QENS results showed that in the coagel phase, only segmental motions are observed, while in gel and fluid phase, both lateral and segmental motions of the lipid take place. In the coagel and gel phases, only a given fraction of the lipid molecules undergo uniaxial rotations, while in the fluid phase, alkyl chains undergo localized translational diffusion. The lateral motion of the lipids, in both gel and fluid phases, could be described by Fick's law, noting that in the fluid phase, the lateral diffusion coefficient is an order of magnitude higher than that in the gel phase.

### C. Additional insights from MD simulations

**FIG. 8.**
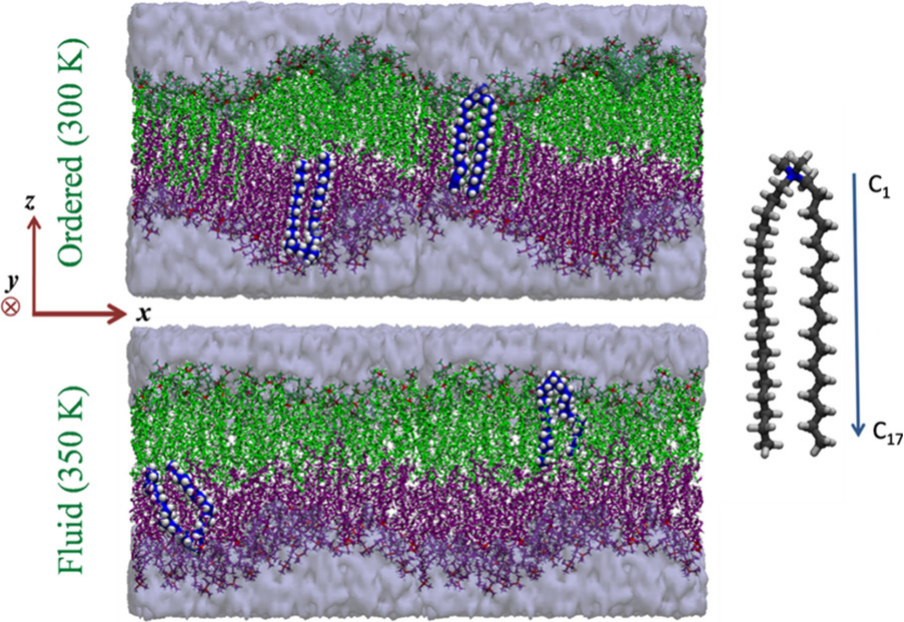
Snapshots of the DODAB lipid bilayer in the fluid and ordered phases. Two DODAB lipids are highlighted in each phase to show the chain conformation in their respective phases. The figure is adapted with permission from Ref. 32. Adapted with permission from Srinivasan *et al.*, J. Phys. Chem. C **122**, 20419 (2018). Copyright 2018 American Chemical Society.

Neutron scattering experiments provide an average picture of dynamics in the DODAB bilayer, which could benefit from a detailed atomistic insight of the various individual degrees of freedom in the system. Their partnership can not only provide a clearer picture of the dynamics but also be useful in refining the models used to describe the experimental data. MD simulations of the aqueous DODAB bilayer system were carried out at two different temperatures, 300 and 350 K. The simulation snapshots of the equilibrated bilayer system at both the temperatures are shown in Fig. 8. At 300 K, an asymmetric ripple phase is formed with significant interdigitation of alkyl chains in some regions, which is similar to the case observed by Jamroz *et al.*^37^ and also observed from SAXS measurements.^36^ However, at 350 K, which corresponds to the fluid phase, no interdigitation of alkyl chains was observed. Based on the converged area per lipid (APL) values, one could recognize that at 300 K (60 Å^2^), the system was more ordered compared to 350 K (64 Å^2^).

#### 1. Alkyl chain structure

**FIG. 9.**
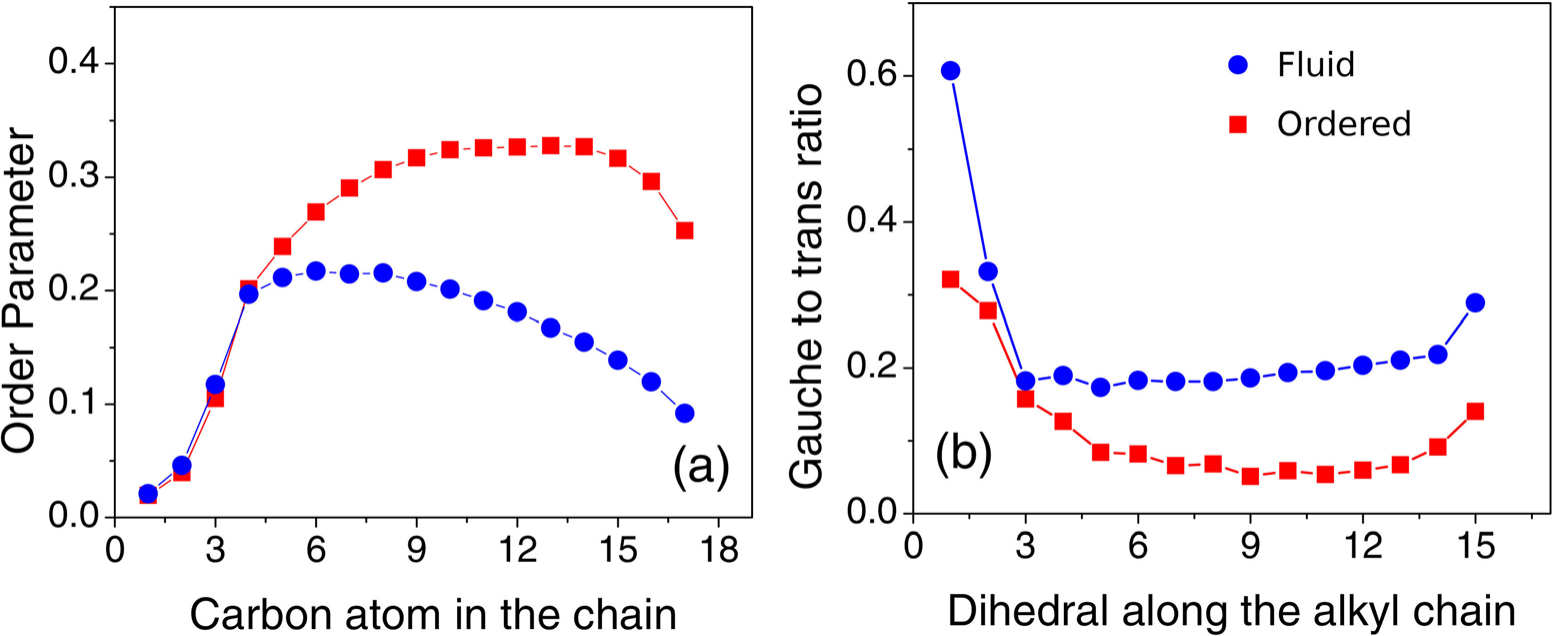
(a) Order parameter (SCH) of the DODAB lipid bilayer calculated for the ordered (squares) and fluid (circles) phases. (b) *Gauche* to *trans* ratio in the alkyl chain of the DODAB lipids in the bilayer in both the phases. Adapted from Dubey *et al.*, Sci. Rep. **8**, 1862 (2018). Copyright 2018 licensed under a Creative Commons Attribution (CC BY) license.

Using Eq. (5), the order parameter (*S_CH_*) of the lipid bilayer was calculated for the bilayer at both temperatures and is shown in Fig. 9(a). It clearly indicates that the bilayer is in a more ordered phase at 300 K compared to 350 K. Henceforth, the system at 300 K shall be referred to as the ordered phase and the system at 350 K shall be called as the fluid phase. The disorder near the headgroup in both the phases can be associated with the molecular architecture of DODAB. In support of the order parameter, the disorder in the fluid phase was also reflected by the increased *gauche* defects. Figure 9(b) shows the *gauche* to *trans* ratio in the DODAB lipid bilayer along the alkyl chain in both the phases. The average *gauche* to *trans* ratio in the fluid phase is ∼2 times larger than in the ordered phase.

#### 2. Lateral motion—sub-diffusive behavior

**FIG. 10.**
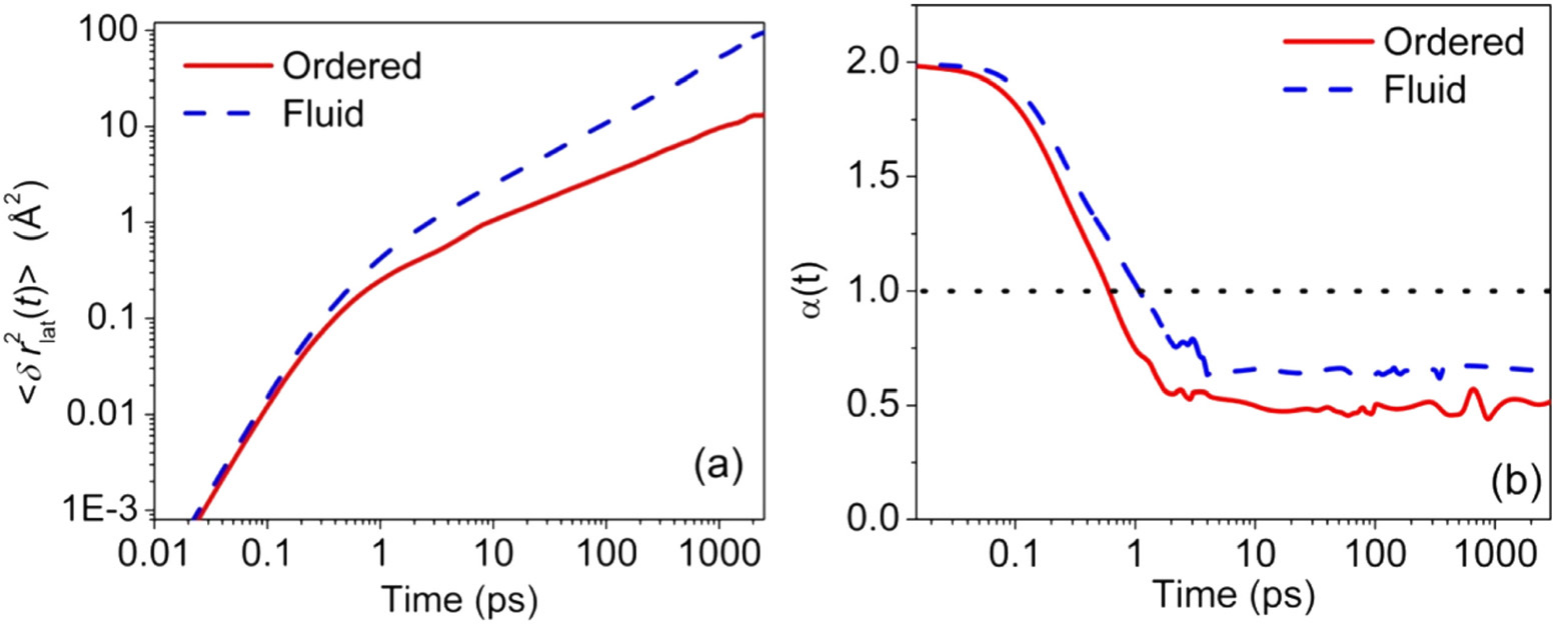
(a) Lateral MSD of DODAB lipids in the ordered and fluid phases. (b) The corresponding subdiffusive exponents for the lipids in both the phases. Adapted with permission from Srinivasan *et al.*, J. Phys. Chem. C **122**, 20419 (2018). Copyright 2018 American Chemical Society.

The mean squared displacement (MSD) of the lipid COM along the x-y plane (bilayer plane) was calculated [based on Eq. (6)] in order to study the lateral motion of lipids along the bilayer. Figure 10(a) shows the plot of lateral MSD for both ordered and fluid phases up to ∼2 ns. It is observed that, at short times, both phases show ballistic behavior with a *t^2^* dependence, which is followed by the subdiffusive regime—described by a power law dependence of *t^α^* (*α* < 1). The explicit dependence of the power law can be calculated using the following formula:

$$\left\langle \delta r_{\mathrm{lat}}^{2}(t)\right\rangle =At^{\alpha }\Rightarrow \alpha (t)=\frac{d\left[\text{ln}\;\left\langle \delta r_{\mathrm{lat}}^{2}(t)\right\rangle \right]}{d\left[\text{ln}\;t\right]}.$$ (17)

**FIG. 11.**
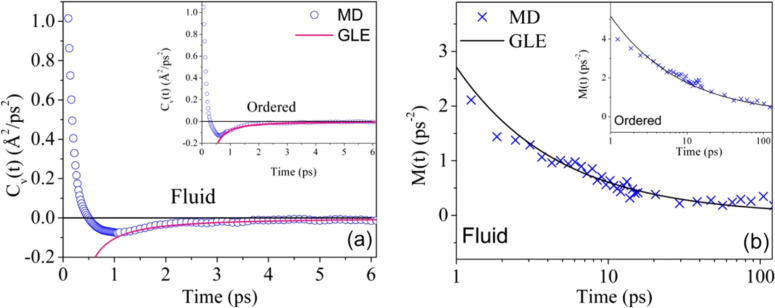
(a) The VACF of DODAB lipids calculated from MD simulation trajectories in the fluid and ordered (inset) phases. (b) The corresponding memory functions calculated from Eq. (21) using the VACF in each phase. The solid lines indicate the theoretical fits based on Eq. (21). Adapted with permission from Srinivasan *et al.*, J. Phys. Chem. C **122**, 20419 (2018). Copyright 2018 American Chemical Society.

Figure 10(b) shows the variation of *α* with respect to time *t*, indicating sub-diffusion in both the ordered (α ∼ 0.5) and fluid (α ∼ 0.62) phases with *α* < 1, wherein a value of *α* = 1 indicates Brownian diffusion. The sub-diffusive motion of lipids in the bilayer can be associated with crowding of lipids in the system, which can be modeled as a non-Markovian diffusion process.^100,101^ The generalized Langevin equation (GLE) with a power-law memory kernel is used to describe the non-Markovian diffusion process of the lipid COM.^102^ For a given particle of velocity **v** in a thermal bath, the GLE is given by^102^

$$\frac{dv}{dt}+\int_{0}^{t}M(t-t\prime )v(t\prime )dt\prime =\zeta (t),$$ (18)

where M(t) is the memory function associated with the diffusion process and ζ(t) is the internal stochastic noise. For the system in thermal equilibrium at a temperature *T*, the memory function and stochastic noise are related to each other through the fluctuation-dissipation theorem,^102^

$$\left\langle \zeta (t).\zeta (t\prime )\right\rangle =\frac{k_{B}T}{m}M(t-t\prime ),$$ (19)

where *m* is the mass of the particle and angular brackets denote the canonical ensemble average. An integrodifferential equation for the velocity autocorrelation function (VACF) of the particle can be obtained from Eq. (18), which is given by^103^

$$\frac{dC_{v}}{dt}+\int_{0}^{t}M(t-t\prime )C_{v}(t\prime )dt\prime =0,$$ (20)

where the VACF, *C_v_*(*t*), for the lipid COM, can be calculated from MD simulation trajectory [Eq. (6)]. Equation (20) can be numerically solved to obtain the memory function associated with the diffusion of the lipid COM. Figures 11(a) and 11(b) show the VACF of the lipid COM calculated from the simulation trajectory and the associated memory function calculated from Eq. (20) for both ordered and fluid phases. In this scenario, where the MSD of the lateral motion obeys Eq. (17), the theoretical asymptotic behavior for the VACF and the associated memory function are given by^103^

$$C_{v}(t)\overset{t\to \infty }{\to }A\alpha (\alpha -1)t^{\alpha -2}M(t)\overset{t\to \infty }{\to }\frac{\left\langle v^{2}\right\rangle }{A}\frac{\;\text{sin}\;(\pi \alpha )}{\pi \alpha }t^{-\alpha }.$$ (21)

The solid lines in Figs. 11(a) and 11(b) indicate the theoretical functions obtained based on fitting the above equations [Eq. (21)] to the calculated values of *C_v_*(*t*) and *M*(*t*) in the asymptotic limit (t > 1 ps), with values of *α* fixed at 0.5 and 0.62 for ordered and fluid phases, respectively. The values of *A* are found to be 0.36 Å^2^/ps^*α*^ (*α* = 0.5) and 0.41 Å^2^/ps^*α*^ (*α* = 0.62) in the ordered and fluid phases, respectively. The excellent quality of the fits indicates that GLE with a power-law memory kernel is a good description of the lateral diffusion of lipids. Based on Eq. (21), the memory function decays faster for a larger value of α, hence suggesting that the memory effects are longer lived in the ordered phase compared to the fluid phase.

While the sub-diffusive behavior of the lateral MSD is described in the framework of the GLE, it is worthwhile to analyze the lateral IISF, *I_lat_*(*Q*,*t*), calculated from MD simulations. In the Gaussian approximation, the lateral IISF can be theoretically written in terms of the lateral MSD, neglecting the higher order cumulants of displacement,

$$I_{\mathrm{Gaussian}}(Q,t)\approx \;\text{exp}\;\left[-\frac{1}{4}Q^{2}\left\langle \delta r_{\mathrm{lat}}^{2}(t)\right\rangle \right]=\text{exp}\;\left[-\frac{1}{4}AQ^{2}t^{\alpha }\right],$$ (22)

where we have used Eq. (17) for the lateral MSD in obtaining the final from for *I_Gaussian_*(*Q*,*t*). The calculated IISF, *I_lat_*(*Q*,*t*), and the associated fits based on *I_Gaussian_*(*Q*,*t*) are shown in Ref. 32 at two typical *Q*-values. The fitting was carried out keeping *α* values fixed at 0.5 and 0.62 for the ordered and fluid phases, respectively. It was observed that the obtained values of *A* match well with their values obtained in the GLE description. Therefore, noting the striking quality of the fits based on the Gaussian approximation, it can be concluded that the lateral dynamics of lipids are spatially homogenous. Hence, the origin of sub-diffusion can be associated with the temporal heterogeneity that is characterized by the memory function in the GLE description.

#### 3. Refinement of the QENS data model

Based on the observation of sub-diffusive nature of lateral motion of DODAB lipids from MD simulations, the dynamical model used to describe QENS data can be refined. In this section, the dynamics of the DODAB lipid bilayer in the fluid phase are modeled and compared with the results obtained from MD simulation and QENS data analysis. The incoherent neutron scattering law is related to the all-hydrogen IISF of the system by a time-Fourier transform. MD simulation trajectories can be used to calculate the all-hydrogen IISF, *I_H_*(*Q*,*t*). The motion of hydrogen atoms is a combination of lateral and internal motions. The QENS data presented until now have been modeled assuming lateral and segmental dynamics; however, MD simulations indicate an extra-dynamical component, faster torsional motions. Considering these three degrees of freedom, the model for fitting the MD simulation IISF can be given as

$$I_{MD}(Q,t)=\text{exp}\;\left(-\Gamma_{\mathrm{lat}}t^{\alpha }\right)\left[a_{0}+\left(1-a_{0}\right)\text{exp}\;\left(-\Gamma_{\mathrm{seg}}t\right)\right]\left[b_{0}+\left(1-b_{0}\right)\text{exp}\;\left(-\Gamma_{\mathrm{tor}}t\right)\right],$$ (23)

where the first term corresponds to the lateral motion, considering spatially homogenous sub-diffusion as described in the framework of the GLE. The second and third terms correspond to segmental and torsional motions, respectively, for which the EISF are given as *a_0_* and *b_0_*, respectively. The characteristic relaxation time of torsional motions (*Γ*_tor_) is ∼1 ps, making it too fast to be observed within the energy transfer range of the IRIS spectrometer. Therefore, the corresponding model for fitting the IISF obtained from the time-Fourier transform of QENS [Eq. (8)] data can be written as

$$I_{\mathrm{QENS}}(Q,t)=\text{exp}\;\left(-\Gamma_{\mathrm{lat}}t^{\alpha }\right)\left[a_{0}+\left(1-a_{0}\right)\text{exp}\;\left(-\Gamma_{\mathrm{seg}}t\right)\right].$$ (24)

**FIG. 12.**
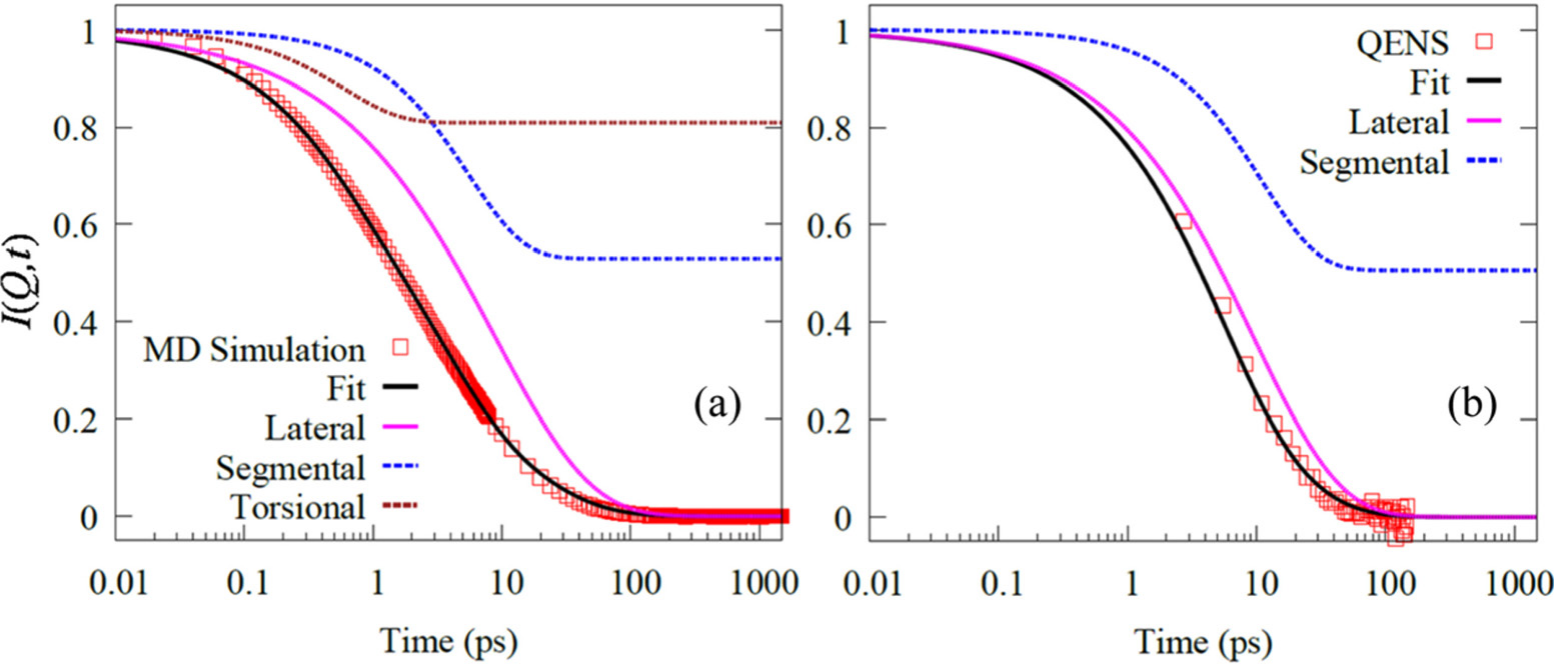
All hydrogen IISF, *I*(*Q*,*t*), from (a) MD simulation trajectories and (b) Fourier transform of QENS spectra at *Q* = 1.2 Å^−1^. The fits based on Eqs. (23) and (24) for MD and QENS are, respectively, indicated along with their respective components. Adapted with permission from Srinivasan *et al.*, J. Phys. Chem. C **122**, 20419 (2018). Copyright 2018 American Chemical Society.

The all-hydrogen IISF calculated from MD simulation (350 K) and QENS data (345 K) at a representative *Q* = 1.2 Å^−1^ are shown in Figs. 12(a) and 12(b). The model fits based on Eqs. (23) and (24) for MD and QENS, respectively, are also shown in the plots, along with the individual components. The quality of the fits indicates that this model successfully describes the dynamics of the membrane in the fluid phase.

**FIG. 13.**
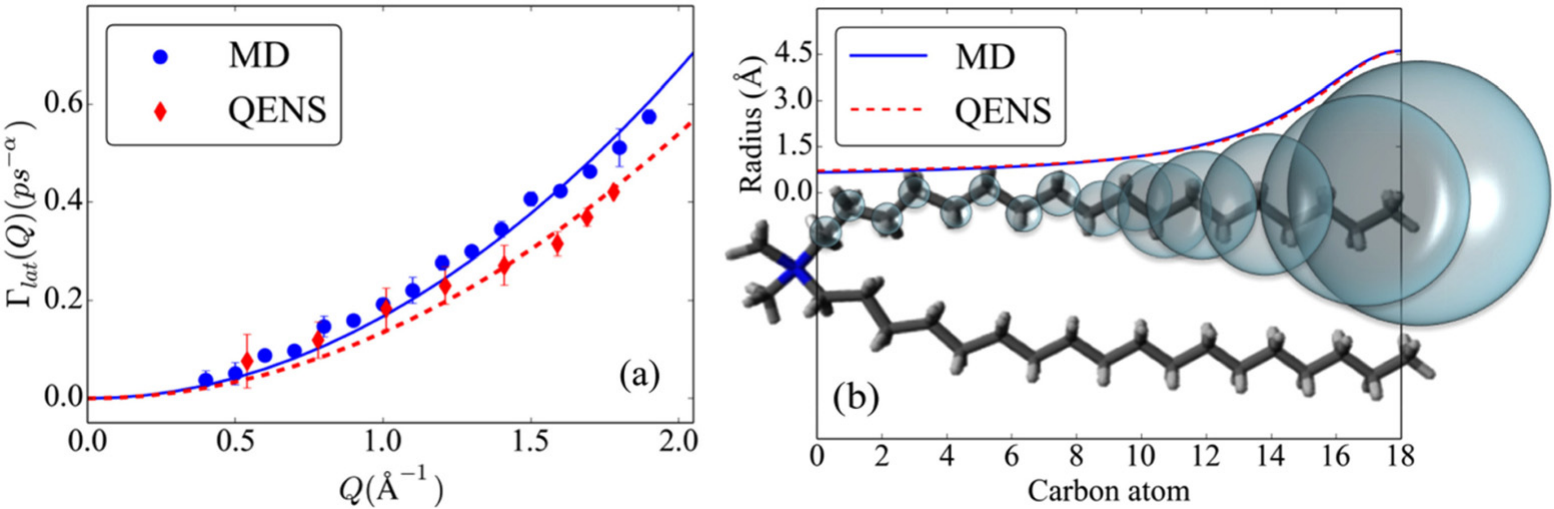
(a) Variation of the lateral HWHM obtained from the same fits. The lines indicate the quadratic fit with respect *Q*. The figure is adapted with permission from Ref. 32. (b) The variation of the radius along the alkyl chain following the half-Lorentzian model (see the text) is shown along with the schematic of a DODAB molecule to show the extent of local diffusion along the lipid tail. Adapted with permission from Srinivasan *et al.*, J. Phys. Chem. C **122**, 20419 (2018). Copyright 2018 American Chemical Society.

The lateral motion of the lipids is characterized by the exponent of sub-diffusion (*α*) and the associated relaxation time (1/*Γ*_lat_). The *Q*-averaged value of the sub-diffusive exponent, *α*, from both the simulation and experiment is ∼0.61, which is very close to the exponent obtained in the GLE description for the lipid COM from MD simulations. The variation of the relaxation timescale associated with lateral motion, *Γ*_lat_, is shown in Fig. 13(a) for both QENS and MD simulation. The solid lines indicate fitting based on the quadratic dependence ¼AQ^2^ [Eq. (22)], assuming a spatially homogenous diffusion process—as observed from the analysis of lipid COM trajectories. The value of *A* obtained from the least-squares fit was found to be 0.42 Å^2^/ps^α^ (α = 0.61) and ∼0.34 Å^2^/ps^α^ (α = 0.61) for MD simulation and QENS experiments, respectively. The obtained values are comparable to their counterpart obtained in the GLE description of the lipid COM.

The MSD of the internal motion of lipid molecules in the membrane indicates that the variation of mobility along the alkyl chain is in fact non-linear along the entire length of the tail.^32^ The segmental mobility is in fact significant only beyond the 10th CH_2_ unit from the headgroup. Instead of the commonly used Carpentier model,^98^ a model in which the variation of radii and diffusivities along the chain to follow a half-Lorentzian distribution is considered. As shown schematically in Fig. 13(b), there is a steep increase in accessible volume in the bottom half of the chain. The obtained values of the EISF, *a_0_*, from MD and QENS indicated excellent agreement between simulation and experiment.^32^ The quality of their corresponding fits,^32^ considering the refined LTD model, is found to be remarkable. The obtained values of *R_max_* from simulation and experiment were found to be equal (∼4.6 Å), and the width of the distribution, *σ_R_*, was also the same (∼3.5). The obtained values of *D_max_* from simulation is ∼20 × 10^−6^ cm^2^/s, while from experiments it was found to be ∼15 × 10^−6^ cm^2^/s. The variation of the radius along the alkyl chain is shown in Fig. 13(b) alongside a schematic of the DODAB molecule. The torsional dynamics of CH_2_ units, observed solely from the fitting of MD simulation results, is found to follow a two-fold reorientation process, with an average residence time of ∼1.4 ps. Clearly, this motion is too fast to be observed with the IRIS spectrometer.

MD simulation results on DODAB lipid bilayers have helped refine the phenomenological models applied to QENS data analysis. A major observation is the subdiffusive nature of lipid's lateral motion, which was successfully explained in the framework of the GLE with a power-law memory function. Subsequently, the subdiffusive nature was also corroborated from the analysis of QENS data of the fluid phase of the DODAB membrane. The insights of individual MSD of the carbon atoms along the alkyl chain of the lipid were useful in obtaining a non-linear heterogeneous model for the variation of radii and diffusivities to describe the segmental motion. The overall conclusions reached from applying both models are qualitatively the same; however, a much more detailed and accurate model is obtained by combining QENS data and MD simulations. In the absence of this partnership and based on QENS data alone, it would be difficult to arrive at such an accurate and suitable description of the lipid molecule dynamical behavior. It, therefore, highlights the importance of this powerful partnership to obtain a more accurate physical description of a system. However, in the absence of any simulation data and based on the statistical accuracy of QENS data, the simpler original model chosen for the QENS analysis [Eq. (12)] is preferred because it involves a smaller number of approximations and fewer fitting parameters.

## IV. EFFECTS OF MEMBRANE ACTIVE MOLECULES ON THE DYNAMICS AND PHASE BEHAVIOR OF DODAB MEMBRANES

**FIG. 14.**
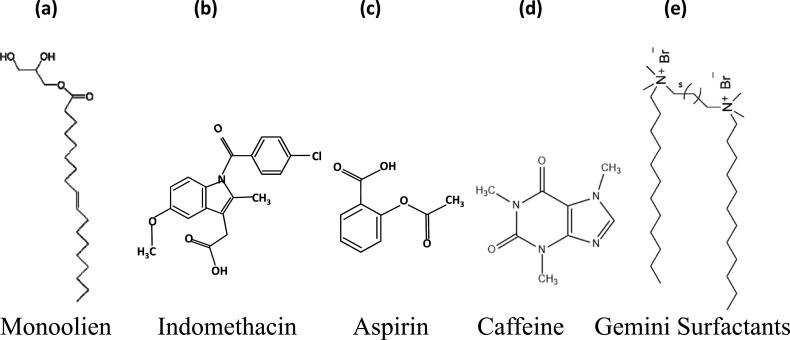
Chemical structures of (a) monoolein, (b) indomethacin, (c) aspirin, (d) caffeine, and (e) gemini surfactants.

With a fundamental understanding of the phase behavior and dynamical characteristics of DODAB membranes, we now review work on changes induced by interacting with a variety of membrane active molecules. Interactions of the DODAB membrane with an unsaturated lipid monoolein, two archetypal nonsteroid anti-inflammatory drugs (NSAIDs), aspirin and indomethacin, caffeine, and gemini surfactants are discussed in Secs. IV A–IV D. For reference, the chemical structure of these molecules is given in Fig. 14.

### A. Effects of monoolein

The maintenance of cell membrane fluidity is of critical importance for various cellular functions including selective permeability, the transport and diffusion of molecules through the membrane, and lipid-protein and protein-protein interactions. In plants and cyanobacteria, at low temperature, when the fluidity of the membrane decreases, these organisms try to compensate this by regulating the number of double bonds in the fatty acids of the lipids. Therefore, it is of interest to investigate how the introduction of unsaturated lipids helps maintain membrane fluidity. In what follows, we discuss the effects of an unsaturated lipid monoolein (MO) on the dynamics and phase behavior of a model cationic lipid membrane made using DODAB. MO is one of the important unsaturated lipids, non-toxic, biodegradable, and biocompatible.^104^ It has widespread applications in the areas of drug delivery, emulsion stabilization, and protein crystallization. DODAB:MO liposomes have been shown to be a potential carrier for gene therapy.^25^ It is found that the presence of MO not only increases DNA's compaction efficiency but also affects the physicochemical properties of the lipoplexes, which can interfere with lipoplex-cell interactions. DODAB:MO formulations showed less toxicity and successfully mediated *in vitro* cell transfection.

**FIG. 15.**
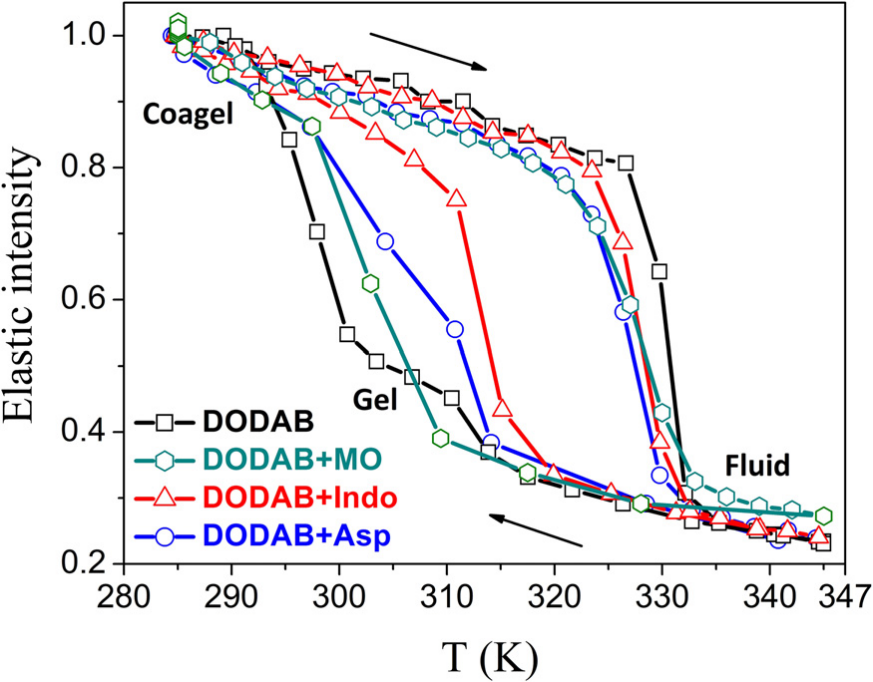
Elastic intensity scans for the DODAB bilayer in the absence and presence of various additives such as monoolein (MO), indomethacin (Indo), or aspirin (Asp) in heating and cooling cycles. Reproduced with permission from Dubey *et al.*, J. Phys. Chem. B **122**, 9962 (2018). Copyright 2018 American Chemical Society. Reproduced with permission from Singh *et al.*, Langmuir **35**, 4682 (2019). Copyright 2019 American Chemical Society.

EFWS and QENS measurements have been carried out on DODAB with and without 33 mol. % MO. Normalized *Q*-averaged elastic intensities as a function of temperature for DODAB in the presence and absence of MO during both heating and cooling are shown in Fig. 15. Incorporation of MO changes the phase behavior of the DODAB membrane significantly in both heating and cooling cycles. Upon heating, the coagel to fluid phase transition is not so sharp and is broader, and the phase transition temperature is lowered (∼321 K), indicating an increase in disorder in the lipid membrane. On the other hand, in the cooling cycle, the formation of the intermediate gel phase is inhibited, and DODAB directly goes from the fluid phase to the coagel phase, indicating synchronous ordering of lipid headgroups and tails. As mentioned earlier, the origin of an intermediate gel phase in the case of pure DODAB is due to the nonsynchronous change in the charged headgroup and hydrophobic tails during cooling. The delayed response of the lipid headgroups compared to the tails on decreasing temperature is due to strong electrostatic interactions between headgroups, in contrast to the weak van der Waals forces in the tails. In the case of the DODAB membrane embedded with MO, it is likely that MO screens the electrostatic repulsion between the cationic headgroups of DODAB and therefore suppresses the formation of the gel phase. The results also show that DODAB:MO complexes still exhibit a large hysteresis (about 10 °C) between heating and cooling.

**FIG. 16.**
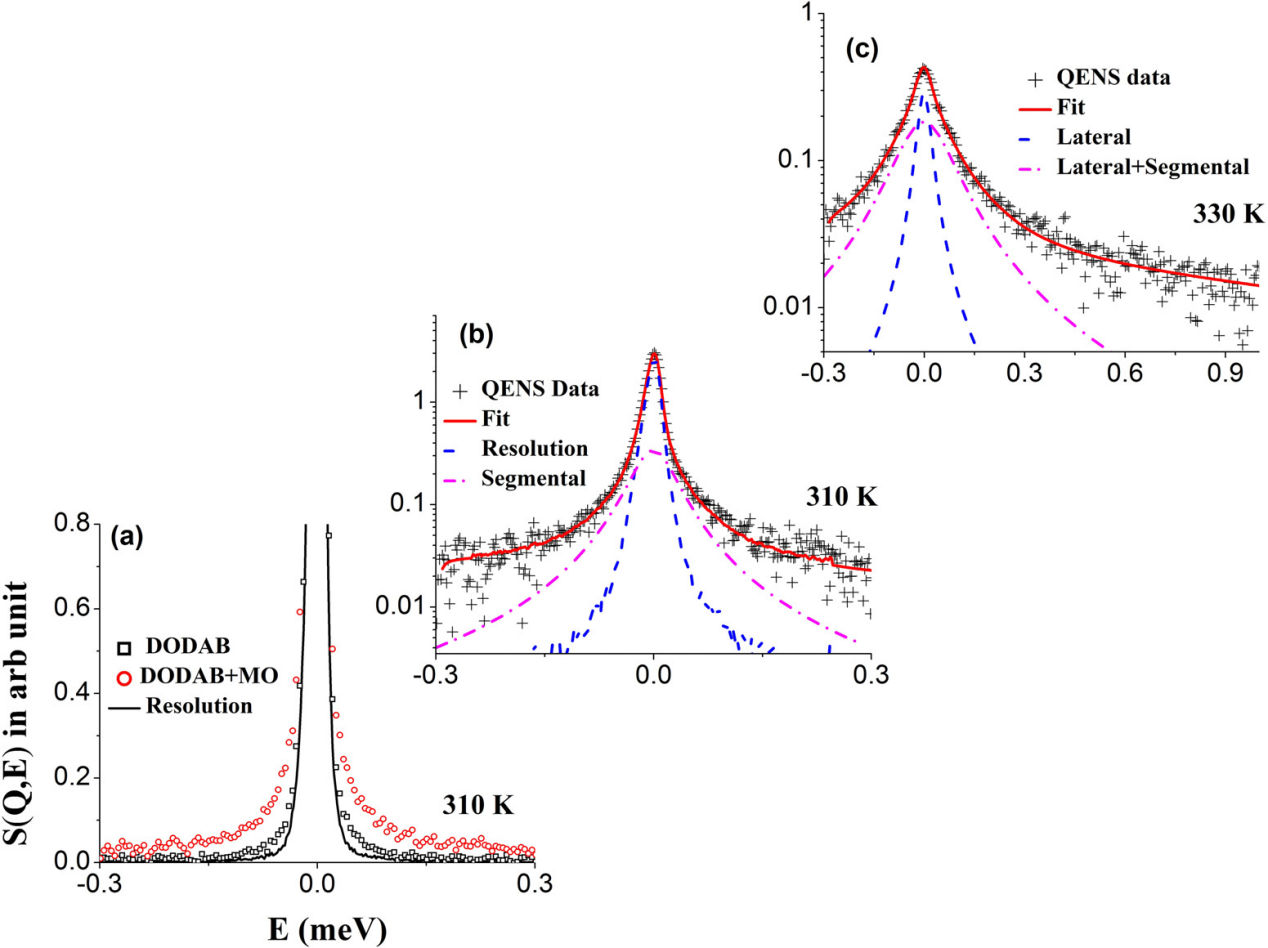
QENS spectra for the DODAB membrane in the absence and presence of MO in the coagel phase (310 K; heating cycle) at a representative *Q* value of 1.4 Å^−1^. The contribution of the solvent (D_2_O) has been subtracted. The instrumental resolution is also shown by the solid line in (a). For direct comparison, all spectra are normalized to the peak amplitude of the resolution. (b) Typical fitted QENS spectra for DODAB in the presence of MO at *Q* =1.4 Å^−1^ in the coagel phase (310 K; heating cycle) and in (c) the fluid phase (330 K). Adapted with permission from Singh *et al.*, Langmuir **35**, 4682 (2019). Copyright 2019 American Chemical Society.

**FIG. 17.**
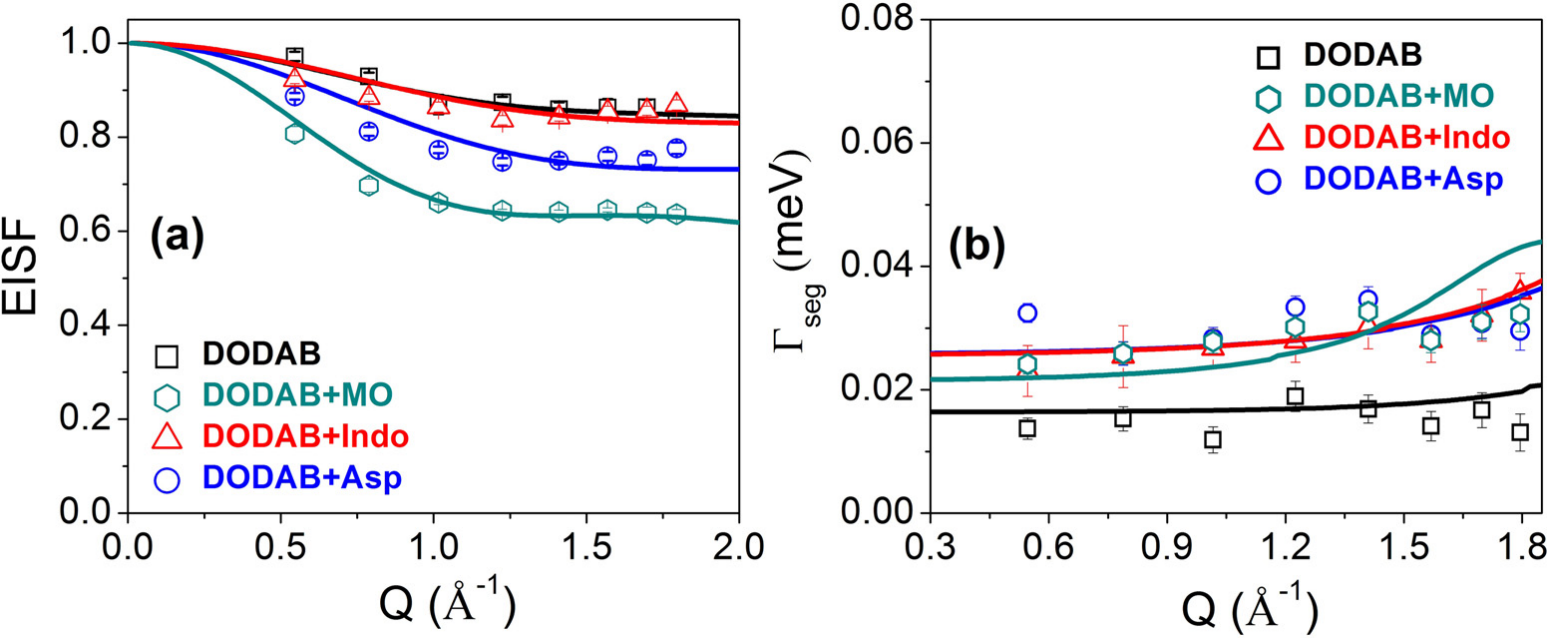
Comparison of the obtained (a) EISF and (b) HWHM, Γ_*seg*_, for the DODAB membrane in the absence and presence of additives such as monoolein (MO), indomethacin (Indo), or aspirin (Asp) in the coagel phase (310 K). The solid lines represent fits assuming the model as described in the text. Reproduced with permission from Dubey *et al.*, J. Phys. Chem. B **122**, 9962 (2018). Copyright 2018 American Chemical Society. Reproduced with permission from Singh *et al.*, Langmuir **35**, 4682 (2019). Copyright 2019 American Chemical Society.

The effect of MO on different lipid motions was probed at 310 K (coagel phase) and 330 K (fluid phase). Representative QENS spectra for the DODAB bilayer with and without MO in the coagel phase (310 K, heating) are shown in Fig. 16(a) at *Q* = 1.4 Å^−1^. In the coagel phase, the incorporation of MO enhances the quasi-elastic broadening, indicating that MO acts as a plasticizer that enhances the dynamics of the membrane. Equation (11) was successfully used to describe the QENS data, as shown in Fig. 16(b). Fitting parameters, the EISF and HWHM (Γ_seg_), for both the systems at 310 K are shown in Figs. 17(a) and 17(b), respectively. Incorporation of MO leads to a decrease in the EISF and to an increase in the quasi-elastic width compared to pure DODAB. This indicates that the addition of MO enhances the conformational flexibility of the lipid molecules and membrane dynamics become faster. Incorporation of MO disrupts the highly ordered and tightly packed lipid structure of the coagel phase, resulting in an enhancement of the flexibility of the lipids. The same fractional URD model that was used to describe the segmental dynamics for the pure DODAB membrane in the coagel phase has been successfully used to describe the dynamics of the DODAB membrane embedded with MO at 310 K, as evident from Fig. 17. The obtained parameters are given in Table I. The presence of MO leads to a large increase in the percentage of mobile hydrogens in the lipid tails, from 13% to 41%. In addition, the radius of gyration (*a*) increases to 2.3 Å from 1.7 Å, indicating more explored volume by the lipid tails. The rotational diffusion coefficient *D_r_* increases from 2.1(±0.3) × 10^10^ s^−1^ to 3.2(±0.3) × 10^10^ s^−1^, and the mean residence time (τ_*MG*_) of methyl group hydrogens decreases from 6.7 ± 0.3 ps to 4.7 ± 0.3 ps. These results show that in the coagel phase, MO acts as a plasticizer, enhancing the dynamics of the DODAB bilayer. The observed results with MO can be correlated with the situation observed in nature where organisms respond to low temperature by incorporating unsaturated lipids via complex mechanisms to enhance the fluidity of cell membranes.

**TABLE I.** Parameters associated with segmental motion of the DODAB lipid in the coagel phase (310 K in the heating cycle) in the absence and presence of membrane active molecules.^43,45,46^

Coagel (T = 310 K)	*p_x_* (%)	*a* (Å)	*τ_MG_* (*ps*)	*D_r_* (×10^10^ s^−1^)
DODAB	13 ± 3	1.7 ± 0.2	6.7 ± 0.3	2.1 ± 0.3
DODAB + MO	41 ± 4	2.3 ± 0.2	4.7 ± 0.3	3.2 ± 0.3
DODAB + Indo	14 ± 3	1.8 ± 0.3	6.0 ± 0.4	3.8 ± 0.4
DODAB + Asp	25 ± 4	1.7 ± 0.2	5.5 ± 0.3	3.6 ± 0.4
DODAB + Caff	16 ± 2	1.9 ± 0.2	6.0 ± 0.3	3.0 ± 0.3

**FIG. 18.**
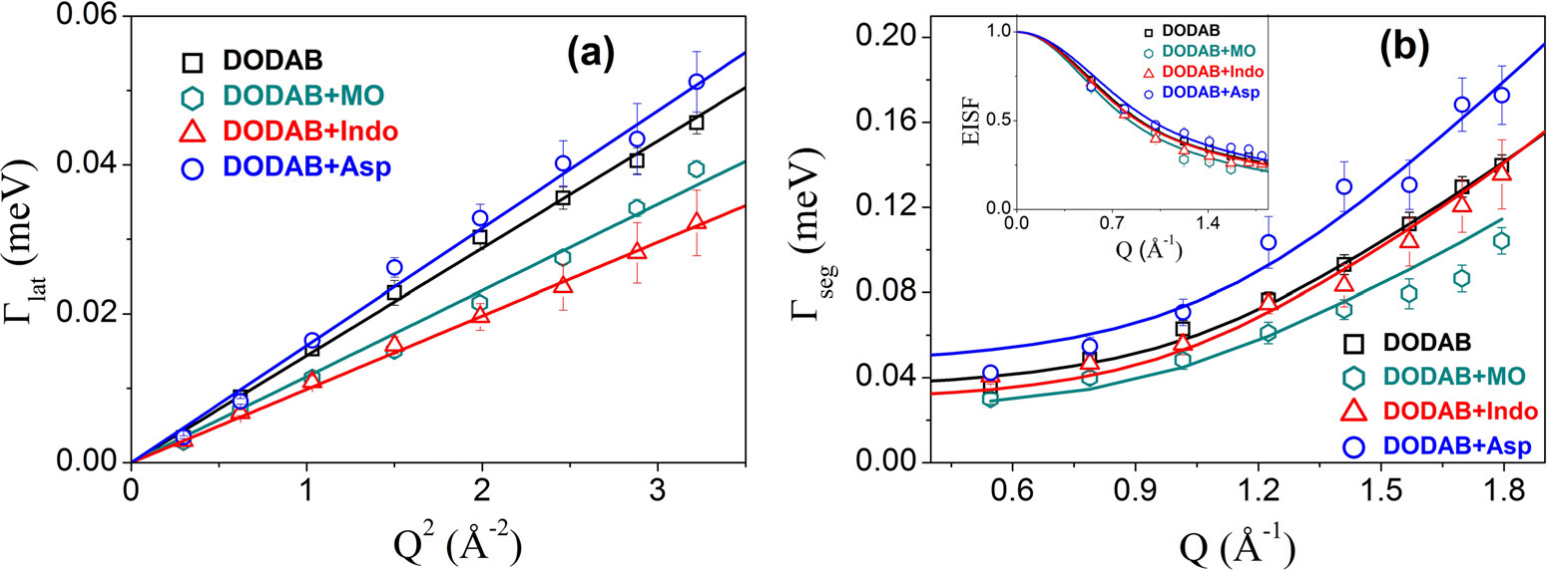
Comparison of obtained HWHMs: (a) Γ_*lat*_ and (b) Γ_*seg*_ for the DODAB membrane in the absence and presence of additives such as monoolein (MO), indomethacin (Indo), or aspirin (Asp) in the fluid phase (330 K). EISFs for the DODAB bilayer with and without MO are shown in the inset. The solid line represents the fit obtained from the models as described in the text. Reproduced with permission from Dubey *et al.*, J. Phys. Chem. B **122**, 9962 (2018). Copyright 2018 American Chemical Society. Reproduced with permission from Singh *et al.*, Langmuir **35**, 4682 (2019). Copyright 2019 American Chemical Society.

Let us now turn to the fluid phase where both lateral and segmental motions of the lipids are observed. As noted earlier, although a refined model was obtained from MD simulation of the pure DODAB membrane, the absence of such simulation data for the DODAB membrane with any of these additives might preclude adopting the refined model. Noting the qualitative equivalence of both the models, the original QENS model [Eq. (12)] was chosen, owing to a fewer number of fitting parameters and was found to describe the data well for the DODAB membrane embedded with MO at 330 K. A representative fitted QENS spectrum is shown in Fig. 16(c). The variation of the resulting HWHM corresponding to the lateral motion (*Γ*_lat_) is shown in Fig. 18(a) and compared to pure DODAB. It is evident that *Γ*_lat_ decreases due to addition of MO and to the effect observed in the coagel phase. In the fluid phase, MO acts as a stiffening agent, restricting the lateral diffusion of the lipid molecules. For the DODAB membrane with MO, the lateral diffusion coefficient, *D*_lat_, decreases to 1.7 (±0.1) × 10^−6^ cm^2^/s from 2.2 (±0.1) × 10^−6^ cm^2^/s. This might be explained by the fact that in the fluid phase, lipid molecules are already disordered with a large area per lipid molecule, and adding MO reduces the available free area per lipid and hinders the lateral diffusion of the lipid molecules. The effect on segmental motions, through HWHM (*Γ*_seg_) and the EISF, is shown in Fig. 18(b). The EISF is almost unaltered due to addition of MO, indicating that the nature of internal motions taking place in the fluid phase does not change much due to the presence of MO. However, *Γ*_seg_ is reduced, indicating a slowing down of the segmental motions. The same LTD model used to describe pure DODAB described the data with MO at 330 K well, as shown by solid lines in Fig. 18(b). The obtained fitting parameters are given in Table II. The size of the spherical domain is 4.5 ± 0.2 Å, similar to that of pure DODAB. However, the diffusion coefficient *D*_max_ decreases from 17.3 (±0.4)) × 10^−6^ cm^2^/s to 14.3 (±0.4) × 10^−6^ cm^2^/s. In summary, the results indicate that the addition of MO restricts both lateral and internal motions of the lipid molecules in the fluid phase.

**TABLE II.** Parameters associated with lateral and segmental motion of the DODAB lipid with and without additives in the fluid phase (330 K).^43,45,46^

Fluid phase (T = 330 K)	Lateral motion	Internal motion		
	D_lat_ (×10^–6^ cm^2^/s)	R_max_ (Å)	D_max_ (×10^–6^ cm^2^/s)	*τ_MG_* (ps)
DODAB	2.2 ± 0.1	4.2 ± 0.2	17.3 ± 0.4	4.5 ± 0.2
DODAB + MO	1.7 ± 0.1	4.5 ± 0.2	14.3 ± 0.4	4.9 ± 0.2
DODAB + Indo	1.5 ± 0.1	4.4 ± 0.3	16.5 ± 0.4	5.5 ± 0.2
DODAB + Asp	2.4 ± 0.1	4.0 ± 0.2	20.4 ± 0.5	4.4 ± 0.2
DODAB + Caff	1.2 ± 0.2	4.9 ± 0.3	13.2 ± 0.3	5.7 ± 0.2

The results from QENS measurements are in agreement with observations from time-resolved fluorescence measurements,^45^ which found that in the coagel phase, addition of MO leads to faster hydration and orientational relaxation dynamics of a fluorophore, but in the fluid phase, it leads to relatively slow hydration and slow orientational relaxation dynamics of the fluorophore. Both methods showed that MO significantly affects the dynamics of the DODAB bilayer and that the effects are strongly dependent on the phase of the lipid bilayer. These effects can be compared to those of another common lipid on the dynamics of lipid membranes, namely, cholesterol. A study on the interaction of cholesterol with four different lipids, two cationic, including DODAB, and two bearing phosphatidylcholine (PC) headgroups, was performed in order to investigate the role of the lipid head group and hydrocarbon chain-backbone linkage.^105^ Measurements showed that the dynamics of the membranes were strongly affected by the inclusion of cholesterol and that the changes were independent of the electrostatic character or head group of the lipid employed. This emphasizes the importance of the hydrophobic interaction between the lipid and cholesterol.

### B. Effects of NSAIDs

Non-steroidal anti-inflammatory drugs (NSAIDs) are well known pharmaceutical formulations prescribed worldwide for their anti-inflammatory, analgesic, anti-platelet, and antipyretic characteristics.^106,107^ In addition to these therapeutic actions, NSAIDs have also shown beneficial effects in treating cardiovascular disease,^108^ cancer,^109^ Alzheimer's,^110^ and arthritis. Although NSAIDs are among the most commonly utilized drugs, their use is associated with a broad spectrum of side effects including gastrointestinal and cardiovascular toxicity.^111^ The action mechanism of NSAIDs^112–115^ has been studied with a variety of methods. The primary target of NSAIDs is the inhibition of cyclooxygenase (COX) activity, which results in the suppression of the production of prostaglandins, the chemical messengers that mediate sensation of pain and inflammation.^116^ However, there is increasing evidence of an alternative action mechanism of NSAIDs mediated by cell membrane-NSAID interactions,^117,118^ owing to the cell membrane being the first biological structure that the drug encounters in its action pathway. This has stimulated the study of the interaction between NSAIDs and membranes, and it has been shown^119,120^ that these interactions are responsible for the NSAID's side effects. This has led to the development of NSAIDs pre-associated with lipids.^115^ Such lipid-NSAID adducts have been shown to significantly reduce side effects and enhance therapeutic activity to inhibit pain and inflammation. In general, it is found that the interaction between NSAIDs and the lipid membrane depends on a number of parameters including the concentration and nature of the drug, ionic strength, and physical state of the lipid bilayer. Recently, it has been shown that in the presence of small proportion of ethanol, DODAB and indomethacin can assemble as nanoparticles that have a very good colloidal stability.^121^ These nanoparticles have potential applications for the *in-vivo* delivery of NSAIDs.

In what follows, we discuss the effects of two commonly used NSAIDs, aspirin and indomethacin, in terms of the changes they induce to the dynamical and phase behavior of the model DODAB membrane, using QENS. Aspirin and indomethacin differ in chemical origin, they are salicylate and indole derivatives, respectively, and their chemical structures are given in Fig. 14. Both the NSAIDs can potentially affect the inflammation responses but at the cost of integrity of the gastrointestinal hydrophobic mucus barrier. To investigate comparatively the effects of these drugs, the same drug to lipid molar ratio was used.

*Q*-averaged elastic intensities for the DODAB membrane in the presence of aspirin/indomethacin as a function of temperature in both heating and cooling cycles are shown in Fig. 15.^43^ It is evident that both drugs significantly affect the phase behavior of the DODAB membrane. In the heating cycle, aspirin and indomethacin shift the coagel-fluid phase transition toward lower temperatures, to 321 and 323 K, respectively. This indicates that incorporation of NSAIDs in the membrane induces disorder, increasing *trans* to *gauche* conformations, caused by the localization of aspirin and indomethacin across the bilayer thickness, which enhances the fluidity of the membrane. These results are consistent with the recent study on NSAIDs on zwitterionic phospholipid membranes,^122^ which also showed a shift in the main phase transition towards a lower temperature. In the cooling cycle, the presence of NSAIDs suppresses the formation of the intermediate gel phase observed in pure DODAB membranes, indicating induced synchronous ordering between the polar headgroups and nonpolar tails of the lipids. This effect is more apparent for indomethacin compared to aspirin. Like MO, NSAIDs must, therefore, screen the electrostatic repulsion between the cationic headgroups, which promotes van der Waals attraction. Both aspirin and indomethacin have pKa values less than five in aqueous solution,^114^ making them anionic at neutral pH. Finally, the data show a reduced hysteresis of around ∼3 °C in the case of indomethacin.

The effects of NSAIDs on the lipid mobility were probed with QENS in both the coagel and fluid phases, at 310 and 330 K, respectively. In the coagel phase, aspirin and indomethacin lead to an enhancement in membrane dynamics.^43^ In fact, the effect of aspirin is stronger. Equation (11) successfully describes the data, indicating that in this phase, only segmental motions are present. The resulting parameter values, *A*(*Q*) and *Γ*_seg_, as shown in Fig. 17 indicate that although the incorporation of the drugs enhances the observed width, only aspirin affects the EISF. Modeling using the fractional URD model gives an increase in the mobile fraction of hydrogen atoms in the lipid chains (*p_x_*) from 13% to 25%, whereas it is almost unchanged for indomethacin. However, the NSAIDs do not affect the radius of gyration. The values of the rotational diffusion coefficient *D_r_* and *τ_MG_* have been extracted from the fractional URD model. For DODAB with aspirin and indomethacin, τ_MG_ is found to be 5.5 ± 0.3 and 6.0 ± 0.3 ps, respectively, compared to 6.7 ± 0.3 ps in pure DODAB. The values of *D_r_* in the case of DODAB + aspirin and DODAB + indomethacin are found to be 3.6 (±0.4) × 10^10^ and 3.8 (±0.4) × 10^10^ s^−1^, respectively, compared to 2.1(±0.3) × 10^10^ s^−1^ in pure DODAB. The observed results indicate that in the coagel phase, incorporation of both NSAIDs enhances the lipid dynamics, resulting in more fluidity of the membrane.

In the fluid phase, Eq. (12) is used to describe the dynamics of the DODAB membrane supplemented with aspirin and indomethacin. Incorporation of indomethacin restricts the lateral motion in the fluid phase, with *Γ*_lat_ significantly reduced compared to the pure DODAB membrane [Fig. 18(a)]. In contrast, aspirin does not affect the lateral motion significantly. Neither aspirin nor indomethacin alters the nature of the lateral diffusion, still following the Fickian law; however, *D*_lat_ is almost comparable or slightly higher in the presence of aspirin, *D_lat_* = 2.4 ± 0.2 × 10^−6^ cm^2^/s, and lower in the presence of indomethacin, *D_lat_* = 1.5 ± 0.1 × 10^−6^ cm^2^/s. In the fluid phase, indomethacin appears to act as a stiffening agent, restricting the lateral motion of the lipid. The reason could be that indomethacin is a larger molecule compared to aspirin with a large hydrophobic part, which penetrates well into the bilayer. This is possibly not the case for aspirin.

The EISF and Γ_seg_ corresponding to segmental motion of the lipid in the DODAB membrane with aspirin/indomethacin are shown in Fig. 18(b) for the fluid phase. The EISFs do not change much due to incorporation of NSAIDs, indicating that the nature of the segmental motions is not altered much for the DODAB membrane in the fluid phase. On the other hand, addition of aspirin leads to an increase in *Γ*_seg_, indicating enhancement in the segmental dynamics of the membrane, while incorporation of indomethacin does not. Modeling using the LTD equation indicates that the explored volume is not significantly affected by the addition of NSAIDs; however, *D_max_* is found to be the largest for DODAB+aspirin, 20.4 ± 0.5 × 10^−6^ cm^2^/s, and least for DODAB+indomethacin, 16.5 ± 0.4 × 10^−6^ cm^2^/s. It is clear that addition of aspirin enhances the internal motions of the lipid, but indomethacin only slightly restricts these. The results from this work can be compared with a recent study on the effect of these NSAIDs on a phospholipid membrane.^122^ In the ordered phase, both drugs enhance the dynamics of the membrane, but the drugs' effects on the membrane dynamics differ in the fluid phase. Indomethacin suppresses the dynamics of the phospholipid membrane, whereas aspirin enhances the dynamics of the membrane. These results indicate that the interaction of NSAIDs with the lipid membrane is a complex interplay between the physical state of the membrane and the nature of NSAIDs. Furthermore, it should be noted that while considering possible effects of administering these drugs, one may need to take into account the influence of NSAIDs on the membrane.

### C. Effects of caffeine

Caffeine (1,3,7-trimethylxanthine) is widely consumed worldwide as a psychostimulant to avoid fatigue or to promote attentiveness.^123,124^ It is a small amphiphilic molecule that is in fact also used as an additive to enhance the pain relief from NSAIDs; however, the exact action mechanism of caffeine is still debatable. Three different mechanisms of action have been discussed in the literature: (i) acts as a pain killer by inhibiting the action of the COX enzyme, (ii) enhances the efficiency of NSAIDs by restraining the oxidation of drugs in the liver, and (iii) inhibits the binding of both adenosine and benzodiazepine receptor ligands to neuronal membrane-bound receptors, which has a positive effect on the frame of mind and performance.^125^ There are additional reports that caffeine interacts directly with the lipid membrane.^125,126^ However, limited information is available on the molecular interactions necessary to understand its action, accumulation, metabolism, and impact on the distribution drugs within a membrane. Recently, x-ray diffraction and MD simulation studies have been carried out on an unsaturated zwitterionic 1-palmitoyl-2-oleoyl-sn-glycero-3-phosphocholine (POPC) membrane with and without caffeine.^125^ The results show that caffeine penetrates into the membrane and mainly locates at the head group–tail group interface of the bilayers. It affects the membrane hydration by attracting water molecules from the membrane to form water pockets around it. Incorporation of caffeine leads to an increase in membrane thickness and an overall decrease in the *gauche* defects in the lipid tails, leading to a decrease in the membrane fluidity.^125^ Effects of caffeine on the 1,2-dipalmitoyl-sn-glycero-3-phosphocholine (DPPC)/dipalmitoyl phosphatidic acid (DPPA) membrane have also been studied in the presence and absence of tetracaine, a local anesthetic compound.^126^ Caffeine reduced the fluidization effects of tetracaine.

The effects of caffeine on the dynamics and phase behavior of the DODAB lipid membrane have been studied using neutron scattering techniques.^46^ The elastic intensity scan shows a similar phase behavior as for pure DODAB. In the heating cycle, the membrane undergoes a coagel to fluid phase transition at 327 K, while in cooling, it goes to the coagel phase via an intermediate gel phase. No effect of caffeine was seen also in DPPC/DPPA membranes.^126^ This is in stark contrast to the effect of MO or the NSAIDs discussed until now and can be ascribed to the difference in charge. Caffeine has an exceptionally high pK_a_ = 14, whereas aspirin, ibuprofen, and indomethacin have pK_a_ values less than five.^114^ Thus, at neutral pH, caffeine will be in a cationic state unlike these NSAIDs. Moreover, caffeine shows a strong affinity towards water.^125^ Therefore, although caffeine remains near the polar region of the membrane, it does not screen the repulsive interactions between charged headgroups and hence does not significantly alter the phase behavior of the DODAB membrane.

Further insights from QENS experiments on the DODAB membrane with and without caffeine suggest that the effects of caffeine on the membrane strongly depend on the physical state of the membrane. In the coagel phase, caffeine slightly increases the quasi-elastic broadening, which suggests an enhancement of the lipid dynamics. The opposite effect is observed in the fluid phase, with a decrease in quasi-elastic broadening, which indicates a stiffening of the membrane. A quantitative analysis showed that in the coagel phase, only segmental motions are present, as Eq. (11) successfully describes the data. The fractional URD model was used to describe the EISF and HWHM of the Lorentzian, and obtained fitting parameters are given in Table I. The incorporation of caffeine leads to a slight increase in the fraction of mobile hydrogens in the lipid tails, from 13% to 16%. In addition, the radius of gyration (*a*) increases slightly from 1.7 Å to 1.9 Å, indicating slightly more flexible lipid tails. The rotational diffusion coefficient *D_r_* increases from 2.1(±0.3) × 10^10^ s^−1^ to 3.0 (±0.3) × 10^10^ s^−1^, and the mean residence time (τ_*MG*_) of methyl group hydrogens decreases from 6.7 ± 0.3 ps to 6.0 ± 0.3 ps.

In contrast, in the fluid phase, both lateral and segmental motions of lipid are observed, as Eq. (12) successfully describes the data. The values of the fitting parameters indicate a restriction in both lateral and internal motions of the lipids: the diffusion coefficient, *D*_lat_, (values given in Table II), decreases by a factor of two, a much stronger effect than seen from the other additives discussed until now. A reason for this significant reduction of lateral mobility might be that caffeine shows preferential hydration, i.e., it removes some of the water molecules from the membrane to hydrate itself, causing dehydration of the membrane and a membrane thickening.^125^ Finally, in terms of the effects on the internal motions, a quantitative analysis was obtained by using Eqs. (15) and (16), and the parameter values are given in Table II. The addition of caffeine again reduces the mobility and stiffens the lipid molecules.

### D. Effects of gemini surfactants

**FIG. 19.**
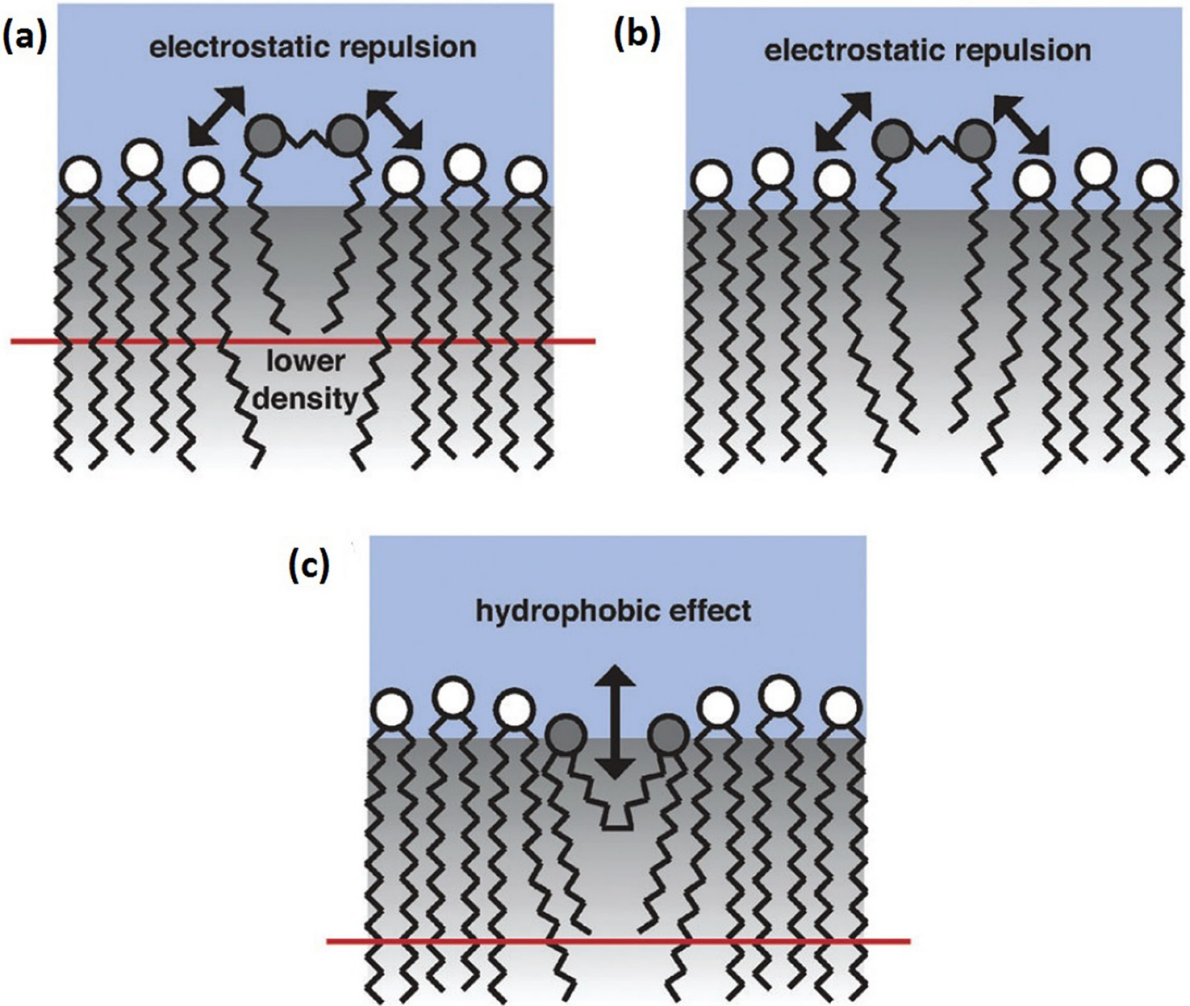
Schematic of the differential vertical positions of gemini polar heads (filled circles) relative to those of DODAB (open circles) considering (a) short tail/short spacer, (b) long tail/short spacer, and (c) short tail/long spacer gemini surfactants. Adapted with permission from Almeida *et al.*, Phys. Chem. Chem. Phys. **13**, 13772 (2011). Copyright 2011 PCCP Owner Societies.

Gemini surfactants are composed of two surfactant molecules bound together covalently by a spacer, as shown in Fig. 14(e).^127,128^ These surfactant molecules, generally represented by *m–s–m*, comprise two ammonium head groups connected by a saturated alkyl chain spacer with *s* carbon atoms and two saturated alkyl tails having *m* carbon atoms. Gemini surfactants have shown promise for skin care, antibacterial regimens, and the construction of high‐porosity materials.^128^ The effect of dicationic gemini surfactants on the phase behavior, structure, and dynamics of the DODAB membrane has also been investigated.^44^ Dicationic gemini surfactants, dimethylene bis(alkyldimethyl-ammonium bromide) and alkylene bis(dodecyldimethylammonium bromide), were synthesized and used as additives in the DODAB membrane. The molar concentration of gemini surfactants in the membrane was varied from 0% to 50%. DSC was used to study the effects of gemini surfactants on the phase behavior of DODAB liposomes.^44^ The main transition temperature of DODAB liposomes decreases with the increasing molar concentration of the Gemini surfactants. For concentrations below 10%, an additional new phase transition peak is observed at a temperature lower than the main transition. The effect of the chain length dependence was also investigated using equimolar liposomes of DODAB and dimethylene bis(alkyldimethyl-ammonium bromide), *m*-2-*m*, where *m* indicates the chain length of the tails.^44^ The main transition temperature was displaced to lower temperatures for *m* = 12 and 16, but for *m* = 18, the peak is broadened and shifted to a higher temperature. Similarly, the effects of spacer length was also studied using equimolar concentrations of DODAB and alkylene bis(dodecyldimethylammonium bromide), 12–*s*–12, where *s* indicates the number of carbon units in the spacer. For the values of *s* = 2, 4, and 6, a systematic decrease in the main transition temperature is observed with the decreasing value of *s*. However, for values *s* = 10 and 12, apart from a decrease in transition temperature with increasing *s*, a set of small peaks are also observed around it, indicating the presence of heterogeneity in the system. The variation of the spacer length has three direct consequences: (i) change in charge density at the level of interface, (ii) modification in the density in the hydrophobic core, and (iii) alteration in the tendency of the hydrophobic spacer to bend toward the interior of the membrane. These three effects together dictate the overall influence of gemini surfactants on the DODAB model system. This non-monotonic behavior can be explained as follows. For small and intermediate spacer surfactants, the disordering effect increases with the decrease in the spacer length due to the increase in charge density in the interface and a reduction of atom density in the deep hydrophobic core. However, for long spacer surfactants, this behavior is altered, and the disordering effect increases with the increase in the spacer length. This is mainly due to the dominance of hydrophobic effects. The spacer bends toward the hydrophobic interior of the membrane, which drags the whole molecule towards a lower vertical positioning that reduces the formation of lower density regions near the core of the bilayer. A schematic showing different vertical positions of gemini surfactant molecules in the DODAB bilayer as a function of spacer and tail length is shown in Fig. 19.

MD simulations of 70% DODAB with 30% gemini surfactants have been carried out in the fluid phase, at 323 K, to get further insights into the structural and dynamical modifications resulting from the addition of gemini. MD simulations were carried out for gemini surfactants, 12–2-12, 12–10-12, and 18–2-18. The order parameter, calculated from these MD simulations, showed a sharp decrease in ordering for the cases of 12–10-12 and 12–2-12 with the former showing a larger decrease in ordering compared to the latter. This strong disorder was also reflected in the calculated mean squared displacement, which was found to increase. On the other hand, the presence of 18–2-18 caused a slight increase in the ordering of lipids in the bilayer, leading to a decrease in the mobility of the lipids. Furthermore, the presence of gemini leads to a decrease in the number of accessible water molecules, as compared to pure DODAB, with 12–2-12 and 12–10-12 showing very significant effects. These studies indicate that the long spacers reduce the charge density on the surface of the liposome, promoting higher disorder in the membrane. However, the longer tail gemini molecules compensate this effect by increasing the density of the membrane in the interior. These results are consistent with the effects of gemini surfactants on phospholipid DPPC membranes.^129^ In the presence of short tail gemini surfactants (12 carbon tails), a decrease in the overall order of the DPPC bilayer was observed. In contrast to this, an increase of order is found in the presence of surfactants with longer tails (16 and 18 carbons).

## V. SUMMARY AND FUTURE OUTLOOK

The molecular dynamics of lipids in the DODAB membrane has been extensively studied across different phases, using neutron scattering experiments and MD simulations. The DODAB membrane exhibits interesting phase behavior, which is dependent on thermal history, i.e., heating and cooling cycles. Each of these phases is characterized by a particular structural arrangement and dynamical mobility of the lipids, which are of great importance in understanding the various aspects and features of more realistic membranes for which DODAB serves as a good model. The elastic fixed window scan (EFWS) and quasi-elastic neutron scattering (QENS) are the two techniques that have been extensively applied to study the dynamics of lipids in the DODAB membrane. While the former provides information on the phase transition associated with the microscopic dynamics, the latter gives a good overview of the main dynamical features of the lipids in the membrane and quantitative parameters to characterize the motions, such as diffusion coefficients, radius of gyration, relaxation times, and volumes explored by the atoms in heads and tails. The response from QENS experiments is an average over all atoms in the system, particularly from hydrogen atoms, owing to their large neutron scattering cross section. Therefore, some of the atomistic details necessary to obtain a complete description of the system are not available. In addition, the statistical accuracy will be a major impediment in our data analysis' ability to differentiate between different physical models. To this end, MD simulations serve as an excellent complementary tool to QENS. They provide atomistic details and hence are useful to refine the models that have been applied in the analysis of QENS data. In parallel, the QENS data serves as a tool to validate the force fields used in the simulations.

EFWS indicates three dynamically distinct phases in the DODAB membrane—coagel, gel, and fluid, in the increasing order of their mobility. The QENS experiments show that in the fluid and gel phases, there are two main characteristic motions that lipids undergo, lateral and segmental motions. As would be expected, the lateral diffusion of the gel phase is about an order of magnitude slower than in the fluid phase, closely related to the structural differences and packing of the lipids in these two phases. In the coagel phase, only segmental motions are observed. Segmental motions can be described as uniaxial rotations in both gel and coagel phases, but a more complex localized translational diffusion model is needed to describe the segmental dynamics in the fluid phase. The insights gained from MD simulation of the DODAB membrane in the fluid phase reveal that the lateral motion is in fact sub-diffusive, with a typical sub-diffusive exponent of ∼0.62. The sub-diffusion of lipids in the membrane is found to be related to the crowding of lipids in the membrane. Furthermore, from MD simulations, the mobility of alkyl chains is found to be more heterogeneous. With the help of these inputs from MD simulation, the QENS data are described with a refined model of the lipid dynamics in the membrane.

**FIG. 20.**
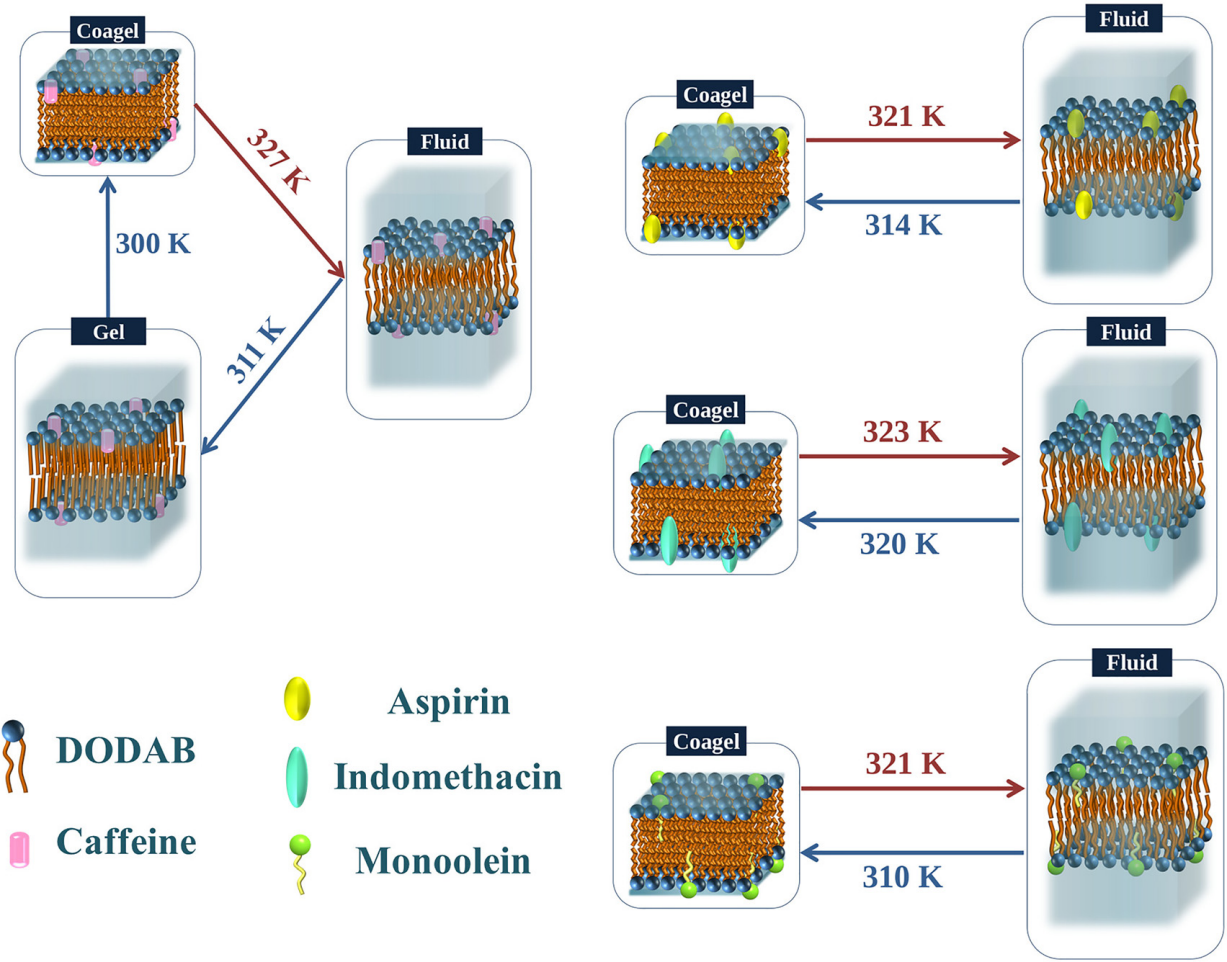
Schematic of the phase behavior of the DODAB bilayer in the presence of different additives (e.g., caffeine, aspirin, indomethacin, and monoolein).

It is clear that lipids do not exist in isolation and in fact the cell membrane is a very complex and multi-component entity. In this review, we survey the interactions of membrane active molecules/additives (e.g., NSAIDs, caffeine, surfactants, and unsaturated lipids) with the lipids in a model membrane made of DODAB lipids and explore their effects on the phase and dynamical behavior of the membrane. The studies conducted to date indicate that the interaction mechanisms between the membrane active molecules/additives and the lipid bilayer are complex and depend on the various factors including the physical state of the membrane and the chemical structure of the additives. It has been shown that the incorporation of these additives induces various degrees of perturbations in the membrane and modulates their dynamical and thermotropic phase behavior. The changes in the phase behavior of the DODAB membrane with different additives are illustrated in the schematic shown in Fig. 20. With the exception of caffeine, all the additives show a significant effect on the phase behavior of the DODAB membrane. In particular, they suppress the formation of the gel phase by screening the strong interaction between the lipid headgroups. Moreover, they also shift both the forward and backward transition temperatures and alter hysteresis significantly. Furthermore, it must be noted that the interactions depend on both the physical state of the membrane and the nature of the additives. In particular, the effects of two NSAIDs, aspirin and indomethacin, at the same molar concentrations show contrasting effects on the membrane dynamics. This suggests that, although NSAIDs may share common features in their chemical pathways of therapeutic action, their interaction with the cell membrane may vary greatly, leading to distinct effects. The chemical structure, size, and hydrophobicity of these additives determine their location within the membrane and, in turn, their effect on the membrane dynamics. The effects of monoolein and caffeine are strongly dependent on the phase of the membrane. In the ordered phase, both behave as plasticizers, i.e., they enhance the membrane dynamics. However, in the fluid phase, both act oppositely, as stiffening agents, by restricting the membrane dynamics. This shows the importance of detailed independent investigations in each of these phases and the need for studies not only on structural changes but also on their dynamical changes.

The dynamics of lipids and its consequences on flexibility of the membrane are key to some physiological functions, and QENS serves as a good experimental tool to characterize such aspects of these systems. MD simulation can provide further insights, in particular about the location and interaction of the additives, which are very useful in understanding their action mechanism. In combination with computational techniques, an accurate description of these membranes can be achieved. As a cationic lipid, DODAB is a known potential candidate for nano-delivery systems to transport gene/DNA, drugs,^21–23,25,26,130^ and a detailed description of the dynamics of the lipid molecules and the effect on the membrane will be of tremendous value to understand their ability as suitable transfection agents. Moreover, the results on the effect of various additives on the lipid dynamics of the membrane might serve as a useful model for DODAB/additive complexes. On that note, it will be of particular interest to study the modulation of lipid dynamics in the presence of additives such as DNA and drugs in the future.

## Data Availability

The data that support the findings of this study are available from the corresponding author upon reasonable request.
